# Evaluating the nationwide availability of essential diagnostics for tuberculosis toward achieving a TB-free India

**DOI:** 10.1128/spectrum.00703-26

**Published:** 2026-08-11

**Authors:** Biswajit Chakraborty, Amaj Ahmed Laskar, Zeeshan Mustafa, Vipin Kumar, Rinki Kumar, Kajal Arora, Anjam Hussain Barbhuiya, Afnan Anowar Hussain, Ashrafun Parvin, Leichombam Mohindro Singh, Kanu Shil, Sumit Roy, Anamika Baruah, Mohammad Azam Khan, Mustafa A. Barbhuiya

**Affiliations:** 1 Foundation for Advancement of Essential Diagnostics, Bio-NEST Bio-Incubation Center, Indian Institute of Technology 28678, Guwahati, Assam, India; 2 Department of Microbiology, Dr. YSP Government Medical College, Nahan, Himachal Pradesh, India; 3 Center for Advanced Laboratory Medicine, Department of Pathology and Cell Biology, Columbia University Medical Center 21611, New York, New York, USA; 4 Department of Paramedical & Allied Health Sciences, School of Medical & Allied Science, Galgotias University 357911, Greater Noida, Uttar Pradesh, India; 5 Department of Zoology, Goalpara College 642130, Goalpara, Assam, India; 6 Microbial Resources Division, Institute of Bioresources and Sustainable Development (IBSD) 63565, Imphal, Manipur, India; 7 Faculty of Paramedical Sciences, Assam Down Town University 497251, Guwahati, Assam, India; 8 District Epidemiologist, Integrated Disease Surveillance Program (IDSP), Karimganj, National Health Mission (NHM), Karimganj, Assam, India; 9 Department of Statistics and Operations Research, Aligarh Muslim University 30037, Aligarh, Uttar Pradesh, India; 10 Department of Pathology, UMass Chan Medical School- Baystate Regional Campus 550083, Springfield, Massachusetts, USA; 11 Department of Healthcare Delivery and Population Sciences, UMass Chan Medical School- Baystate Regional Campus 550083, Springfield, Massachusetts, USA; End TB Dx Consulting LLC, San Diego, California, USA

**Keywords:** diagnostic access, LMICs, tuberculosis, public health, multidrug resistance, WHO Essential Diagnostics List, infectious diseases, laboratory medicine

## Abstract

India continues to face a high tuberculosis (TB) burden, as reaffirmed by the latest World Health Organization (WHO) Global TB Report. This trend highlights the need for a critical appraisal of the country’s TB management framework. In this study, we assessed the distribution of TB Treatment Units (TUs) and Culture and Drug Susceptibility Testing (CDST) laboratories across states and union territories in India. The analysis revealed a pronounced national deficit of 61.56% in CDST laboratory capacity relative to projected diagnostic needs, with the gap most evident in rural and geographically remote regions. Lack of proportional availability of drug susceptibility testing or alternative molecular methods in conjunction with TB Treatment Units, leading to non-selective antibiotic use, could be the reason India has the highest multidrug-resistant TB. Our multi-state assessment of 327 healthcare facilities across seven states and the National Capital Region-Delhi revealed marked deficiencies in the implementation of essential diagnostics (ED) for tuberculosis in primary-level public health care facilities. Notably, access to molecular diagnostics, such as nucleic acid amplification tests (NAATs), remains insufficient for early case detection, contributing to a high proportion of missed TB cases. The key TB treatment management and monitoring investigations, such as comprehensive metabolic panels (CMP) and complete blood counts (CBC), are inconsistently available at TB Units linked to Primary and Community Health Centers. Addressing these systemic deficiencies through targeted policy action is critical to integrating essential diagnostics into routine care pathways and ensuring an accessible, high-quality TB elimination program.

**IMPORTANCE:**

The study analyzes the Government of India’s tuberculosis (TB) Free India by 2025 campaign in the context of the availability of essential tuberculosis diagnostics. Our findings raise the question of why India may not achieve the ambitious goal of eliminating tuberculosis, perhaps by 2030, in the absence of a proportionate and balanced TB diagnostic toolkit. To date, a comprehensive study of the availability and access gap for the World Health Organization's Essential Diagnostics List (EDL) in India is lacking in the scientific and public health discourse.

## INTRODUCTION

Tuberculosis (TB) remains a major public health challenge globally and in India (1, 2). According to the World Health Organization Global TB Report 2024, India accounts for nearly 25% of the global TB burden (2). Despite ongoing national efforts, substantial gaps persist between estimated and reported TB cases worldwide and within India (2). The Global TB Report 2025 estimated a gap of approximately 2.4 million TB cases globally in 2024, with India contributing 8.8% of these missing cases, second only to Indonesia (10%) (2). Furthermore, the National TB Prevalence Survey of India (2019–2021) reported that approximately 31% of individuals aged 15 years or older have TB infection (3). Although government reports suggest a decline in missing TB cases in India from 1.5 million in 2015 to 0.1 million in 2024 (3), recent global estimates indicate that underdiagnosis remains a significant concern (3). A critical yet often unrecognized factor contributing to the persistent disease burden is the unequal distribution of diagnostic services across India’s diverse populations and geographies (4, 5). Access to TB diagnostics varies widely between urban and rural areas, as well as among socioeconomically disadvantaged and marginalized communities, including tribal populations, remote northeastern regions, and urban slums (6, 7). Inadequate infrastructure, shortage of trained personnel, and uneven deployment of advanced diagnostic tools, such as nucleic acid amplification tests (NAAT)/cartridge-based nucleic acid amplification tests (CBNAAT), further widen these gaps (8). Consequently, populations in underserved settings frequently experience delayed diagnosis or remain undiagnosed, sustaining transmission and worsening disease outcomes. This inequitable access to essential TB diagnostics is a key structural driver of India’s high TB burden and continued gaps in case detection (9). Another major contributor to India’s TB burden is the increasing prevalence of drug-resistant TB. India accounted for 32% of global multidrug-resistant/rifampicin-resistant TB cases in 2024 (10, 11). This is likely driven by undiagnosed and untreated resistant cases, leading to poor treatment outcomes and increased mortality in certain populations (11). To strengthen TB control, the Government of India has implemented several initiatives under the National Strategic Plan, including a web-based, real-time, case-based digital tracking system (12). Additionally, the National Tuberculosis Elimination Program (NTEP) places TB Units at the core of service delivery, supported by Culture and Drug Susceptibility Testing (CDST) laboratories for diagnosis of TB, drug resistance, and relapse (12, 13). Strengthening these laboratory networks is critical for early detection of drug-resistant TB, which remains a key bottleneck (13, 14). Globally, the World Health Organization (WHO) introduced the Essential Diagnostics List (EDL) in 2018 to improve access to quality diagnostics (15). In alignment with this, India launched the National Essential Diagnostics List (NEDL) in 2019, prioritizing TB diagnostics (16). India’s healthcare system operates through a tiered structure comprising primary (sub-centers, primary health centers), secondary (community health centers, sub-district hospitals), and tertiary care (district hospitals and medical colleges) (17). Under NEDL-2019, essential TB diagnostics include acid-fast bacilli (AFB) smear microscopy, chest X-ray, nucleic acid amplification tests (NAAT/CBNAAT), and fluorescence microscopy (16). WHO further recommends rapid molecular diagnostics, such as NAAT/CBNAAT, as first-line tests for early and accurate TB detection (18). However, in India, these high-sensitivity tools are largely limited to secondary and higher-level facilities, resulting in only about 33% of TB cases being diagnosed using such methods (19). India also continues to face challenges related to TB relapse (approximately 27.1%) and mortality (approximately 5.6%) (20, 21). These issues may be linked to a lack of awareness of follow-up diagnostic tests, such as the complete blood count (CBC) and complete metabolic panel (CMP), required for monitoring a TB patient’s treatment. These two panels of clinical tests are important for assessing nutritional status, liver function, anemia, and pancytopenia in patients receiving several antitubercular drugs. Monitoring is particularly critical for patients with drug-resistant TB, who require closer clinical and laboratory follow-up (22). Despite multiple policy initiatives, the persistently high TB burden highlights the need to reassess the implementation of diagnostic and management strategies at all levels of the healthcare system. Previous studies based on the WHO EDL across multiple countries, as well as limited studies from Indian states, have identified significant gaps in diagnostic availability, particularly at the primary care level (23, 24). However, a comprehensive national-level assessment of TB-related essential diagnostics under India’s NEDL is lacking. This study evaluated the current implementation status of TB management in India by assessing access to TB Units, CDST laboratories, TB-specific essential diagnostics, and diagnostic tests required for treatment monitoring, including CBC and CMP.

## MATERIALS AND METHODS

### Design of the study

To examine potential gaps in the health system, we conducted a comprehensive assessment of the TB diagnostic and management infrastructure nationwide. In the first phase, we extracted state-wise data on the number of TB Treatment Units (TUs) and CDST laboratories from official publicly available government databases. The TB Treatment Units are referred to in this manuscript as TB Units (TUs). In the subsequent phase, to assess the basic infrastructure supporting TB management, we designed a field-level survey to evaluate the availability of essential diagnostics as per the NEDL-2019 across different tiers of government health facilities in select states and districts. The selection of survey sites was based on random sampling and guided by operational feasibility and resource availability.

### Collection of data regarding the distribution of TB Units across India

Information on the number of TB Treatment Units (TUs) was obtained from the Government of India’s Ni-Kshay portal. Ni-Kshay (Ni means “End”; Kshay means “Tuberculosis” in Hindi) is the web-enabled patient management system for TB control under the NTEP. The district-level data on functional TB Units were collected and then aggregated to calculate the total number of TB Units for each state and union territory (UT) (25). A customized map was created using an online tool (www.iipmaps.com) to visualize the current distribution of TB Units across India (26). The number of recommended TB Units for each state and union territory was calculated in accordance with the NTEP guideline of one TB Treatment Unit per 0.2 million population, using the most recent population estimates available on the Statistics Times website as of 5 February 2025 (27). Finally, the gap in TB Units was calculated as the difference between the existing and recommended numbers, and this information was displayed for each state and UT on a customized map of India. The details of the existing and recommended TB Units for each state and UT are presented in Table S1.

### Assessment of the distribution of CDST-based TB diagnostics across India

The WHO recommends that at least one CDST laboratory be available for every five million people to ensure effective TB management (28). In this study, information on the availability of CDST laboratories across India was collected using data from the India TB Report 2024 (1). The current population (in millions) for each state and union territory was obtained from the Statistics Times (www.statisticstimes.com) website (27). Gaps in CDST laboratory infrastructure coverage were then calculated using the following equations:


(1) $$Recommended\;number\;of\;CDST\;labs=\frac{Population\;\left(in\;millions\right)}{5}$$

(2) Availability Gap in CDST=Recommended CDST−Existing CDST

(3) $$\begin{array}{rl} \text{ CDST Labs:TB Unit Ratio (Recommended/Existing) } & \\ =\frac{\;\;\;Recommended\;or\;Existing\;number\;of\;CDST\;Labs}{Recommended\;or\;Existing\;number\;of\;TB\;Units} \end{array}$$

The detailed state- and UT-wise numbers are presented in Table S1, showing India’s current population, the existing number of CDST laboratories, the WHO-recommended number of CDST laboratories, and the gaps in CDST laboratory facilities.

### Survey of TB essential diagnostics in different categories of public healthcare settings

**Fig 1:**
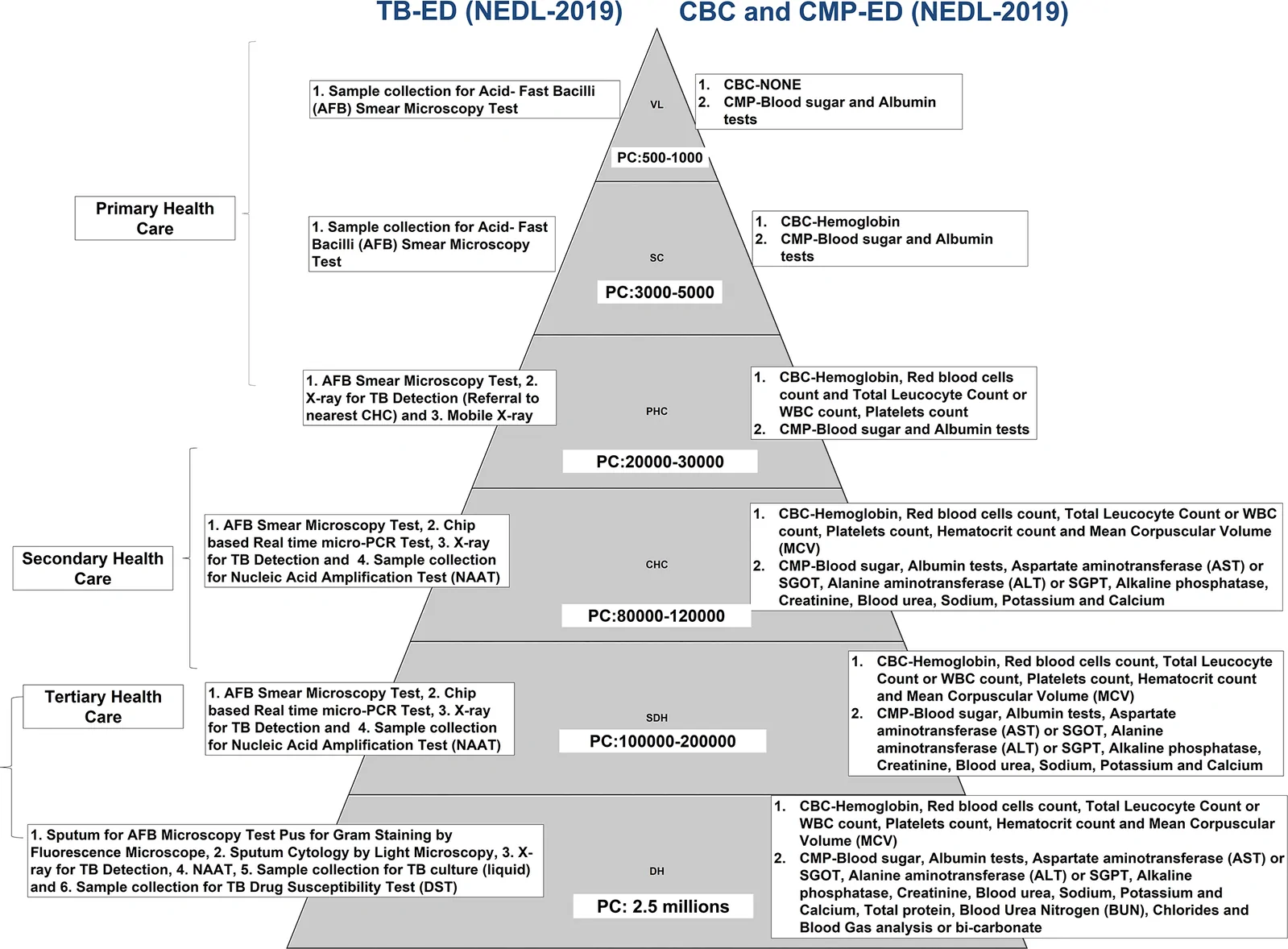
Hierarchical structure of the Indian healthcare system showing primary, secondary, and tertiary levels of care. The primary level includes village level (VL), sub-center (SC), and primary health center (PHC). The secondary level includes the community health center (CHC) and sub-district hospital (SDH). The tertiary level includes the district hospital (DH). The left side of the pyramid lists the TB ED recommended under the NEDL-2019 for each healthcare level. The right side of the pyramid lists the CBC and CMP tests recommended under NEDL across the respective levels of care. The corresponding population coverage (PC) for each level of healthcare is provided as a number in the respective tier; for example, at the VL, the PC is 500–1,000.

In this study, the availability of essential diagnostics (ED) for TB screening and diagnosis (Fig. 1) along with TB treatment monitoring diagnostic tests (Fig. 1), was assessed through surveys across five districts in Assam and Uttar Pradesh (UP), four in Tripura, three in Delhi, three in Manipur, and one district each in Himachal Pradesh (HP), Haryana, and Rajasthan. The surveyed sites are shown in deep black color in Fig. S1. Although the study sites were selected primarily based on operational feasibility and the availability of local collaborators, efforts were also made to ensure geographic diversity by including remote North-Eastern states, densely populated northern regions, and the metropolitan territory of Delhi. This broad representation provides valuable insights into the current status of healthcare infrastructure, diagnostic capacity, and access to essential diagnostic services across diverse healthcare settings in India. The survey focused on the availability of essential diagnostic tests for TB across different categories of healthcare facilities, as recommended by the Indian Council of Medical Research (ICMR) in the NEDL. A village-level survey was not conducted because the NEDL (2019) recommendations are the same for both village-level facilities and sub-centers (Fig. 1). Additionally, during the initial phase of the study, most village-level healthcare facilities were inaccessible at the time of the survey.

Although the ICMR released the second version of NEDL in 2025, this survey was conducted using the first version, NEDL-2019, before its release (16, 29). The survey was conducted through physical visits to public healthcare facilities between November 2023 and April 2025. It was designed as a cross-sectional study at a single time point. Facility staff were interviewed using structured questionnaires (Tables S1 and S2). The presence or absence of diagnostic tests across different categories of healthcare facilities was recorded as an indicator of diagnostic service availability. It is important to note that staffing data were initially collected; however, because laboratory technicians are often shared across multiple facilities, which would have resulted in a complex, difficult-to-interpret data set, the current study focused on the availability of diagnostic services as the primary outcome. The presence of diagnostic services was considered indicative of functional service delivery, as test performance and result validity are under the purview of the Medical Officer of the respective facility.

Overall, this survey assessed the availability of essential TB diagnostics across seven Indian states—Assam, Manipur, Tripura, HP, UP, Rajasthan, Haryana—and one union territory, Delhi. The districts within the different states that were covered included Cachar, Sribhumi, Hailakandi, Goalpara, and Kamrup Metro (Assam); South Tripura, North Tripura, West Tripura, and Unakoti (Tripura); East Imphal, West Imphal, and Bishnupur (Manipur); Sirmaur (HP); Gautam Budh Nagar, Aligarh, Budaun, Bulandshahr, and Kasganj (UP); Jaipur (Rajasthan); Faridabad (Haryana); and Central Delhi, South West Delhi, and New Delhi (Delhi) (Fig. S1). In total, we surveyed 327 public health facilities, comprising sub-centers (SCs, *N* = 76), primary health centers (PHCs, *N* = 150), community health centers (CHCs, *N* = 78), and district hospitals (DHs, *N* = 23). Most of these districts and survey sites in the north-eastern states are remote and difficult to access. The successful implementation of the survey in these areas was largely facilitated by local collaborators, who played a crucial role in securing access. This demonstrates that, while such locations are challenging, they are not entirely inaccessible when appropriate local engagement is in place. This local presence and familiarity with the terrain and communities significantly contributed to the feasibility of surveying these settings. Other sites in New Delhi, UP, Rajasthan, and Haryana served to reduce selection bias and provide representative data for a snapshot of TB diagnostic facilities in India.

### Statistical analysis

The study was conceived to provide the first representative, ground-level assessment of the implementation status of TB-management tools across India, using state- and UT-wise data generated within the constraints of our operational resources. Accordingly, surveys were undertaken only in locations where logistical support was available, rather than to achieve uniform state-wise comparability. Consequently, the extent of coverage varied: in some states, only a single district could be surveyed, whereas in others up to five districts were included. Likewise, while certain states’ assessments covered all four categories of health-care facilities, in others, we were able to survey only two categories. The number of facilities assessed within each category also differed considerably. These inherent asymmetries and logistical or operational biases limited the scope for robust statistical comparisons across multiple data sets. However, inferential statistics play a vital role in drawing meaningful conclusions from sample data and making generalizations about the target population. A normality test is used to assess whether a data set follows a normal distribution, an important assumption for many statistical methods, such as *t*-tests and ANOVA. The main purpose of this test is to decide whether these methods are suitable for the data. In this study, two normality tests are used: the Shapiro–Wilk Test and the Kolmogorov–Smirnov Test. In general, the null hypothesis states that the data are normally distributed. If the *P* value is less than the chosen significance level (usually 0.05), it suggests that the data do not follow a normal distribution. In this study, both parametric and non-parametric statistical tests are employed to ensure robustness and reliability of the results. For parametric analysis, the assumption of normality is examined, and, where satisfied, an independent-samples *t*-test is applied to compare group means. When the normality assumption is violated or when data are ordinal, appropriate non-parametric alternatives, including the Mann–Whitney *U* test and the Kruskal–Wallis test, are used to assess group differences. Furthermore, the chi-square test is employed to examine associations and assess the significance of differences between observed and expected frequencies in the data. The combined use of these statistical techniques enhances the validity of the findings and ensures that the study objectives are addressed rigorously and comprehensively.

## RESULTS

### Critical availability gaps in TB Units and CDST laboratories across Indian states and UTs

**Fig 2:**
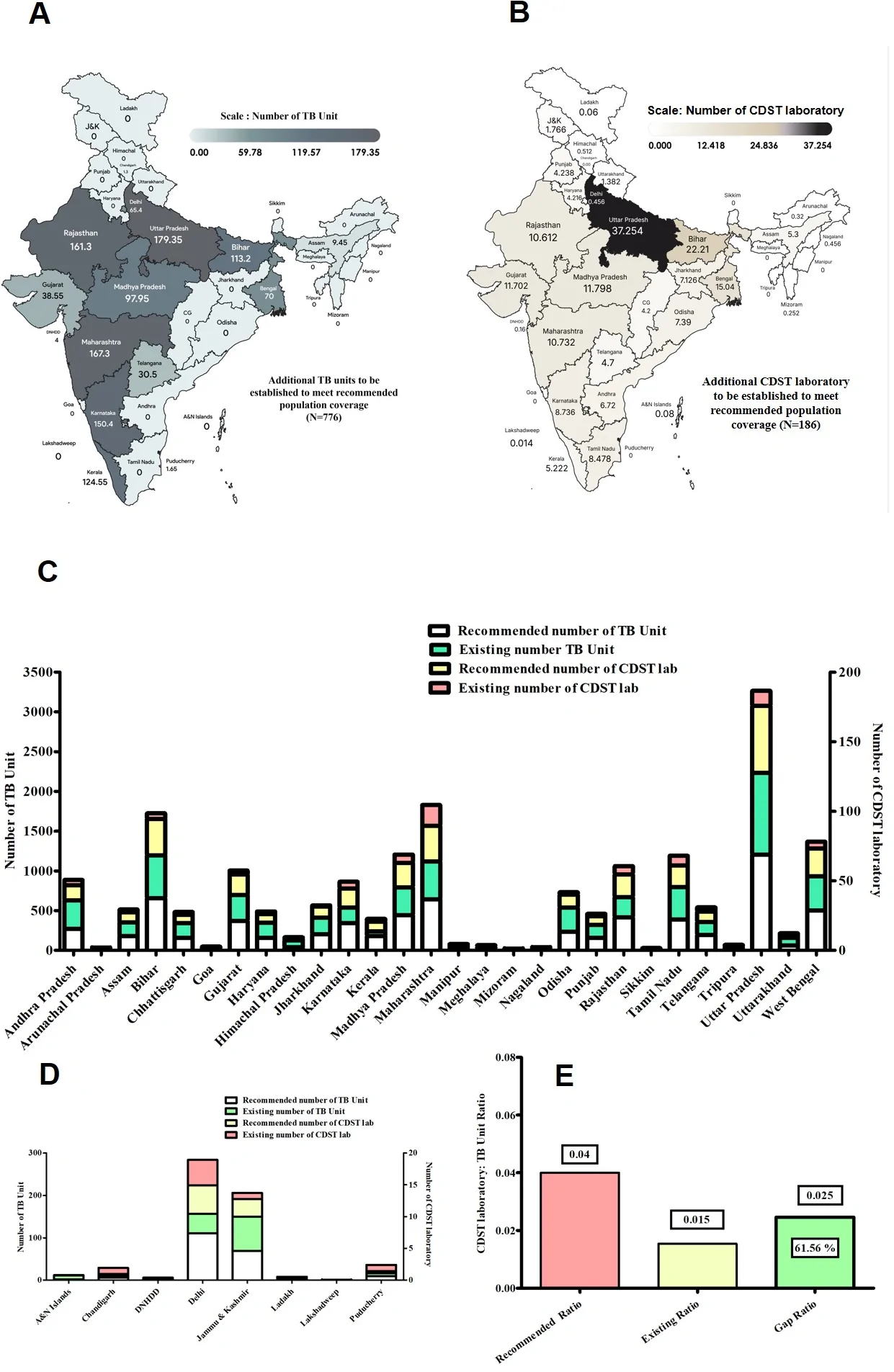
Gaps in the number of TB Units and CDST laboratories across Indian states and UTs are shown using customized maps. (**A**) TB Units: the national gap of 776 TB Units was calculated as the difference between the recommended (7,084) and existing (6,308) units, with state- and UT-wise gaps shown (Table S1). (**B**) CDST laboratories: the national shortfall of 186 laboratories was estimated by subtracting existing facilities (97) from the recommended (283), with corresponding state- and UT-wise gaps (Table S1). (**C**) State-wise analysis: existing TB Units were obtained from the national TB reporting portal, and recommended numbers were calculated using the NTEP norm of one TB Unit per 200,000 population (population data as of 5 February 2025). Existing CDST laboratory data were obtained from the (30) India TB Report 2022, and recommended numbers were estimated using WHO guidelines (one CDST laboratory per 5 million population). Chi-square analysis showed significant differences between recommended and existing TB Units (*P* = 0.0001; *χ*² = 566.93; df = 27) and CDST laboratories (*P* = 0.001; *χ*² = 144.44; df = 27). (**D**) UT-wise analysis: the same methodology was applied for union territories. Significant differences were observed for TB Units (*P* = 0.0001; *χ*² = 73.43; df = 6), while no significant difference was found for CDST laboratories (*P* = 0.66; *χ*² = 4.95; df = 6) (Table S1). (**E**) National CDST laboratory-to-TB Unit ratio: the existing ratio was calculated from observed facility numbers, while the recommended ratio (1 CDST per 25 TB Units; 0.04) was derived from guideline-based estimates. The percentage gap was calculated by comparing existing and recommended ratios.

Ensuring sufficient TB Units and CDST laboratories is essential for effective TB control in India. Based on national benchmarks—one TB Unit per 200,000 population (NTEP) and one CDST laboratory per 5 million population (WHO)—we evaluated the existing infrastructure across all states and UTs. India currently has 6,308 TB Units against the required 7,084, creating a deficit of 776. Eleven states and four UTs fall below the recommended numbers, with the largest gaps in Uttar Pradesh (179), Maharashtra (167), Rajasthan (161), Karnataka (150), Kerala (125), and Bihar (113). In total, deficit regions require 1,253 additional TB Units to meet the benchmark (Fig. 2A). For CDST laboratories, India has 97 (public and private) but needs 283, resulting in a shortfall of 186. Nineteen states—including Bihar (22), Uttar Pradesh (37), West Bengal (15), Gujarat (12), Madhya Pradesh (12), Rajasthan (11), Maharashtra (11), and Odisha (7)—and one UT (Jammu and Kashmir, 2) fall below the requirement (Fig. 2B).

### Availability gaps in CDST laboratory to TB Unit ratio across India

Effective TB management depends on the combined functioning of TB Units and CDST laboratories. To assess their relationship, we compared the existing numbers for each state and UTs. States showed wide statistically significant variation in both TB Units and CDST laboratories (Fig. 2C). However, statistically significant variability was observed among UTs for TB Units (*χ*² = 73.43, df = 6, *P* value = 0.0001), but a non-significant difference was observed among UTs for CDST laboratories (*χ*² = 4.95 with df = 7, *P* value ≈ 0.66) (Fig. 2D). State- and UT-wise details on the number of TB units and CDST laboratories are provided in Table S1. According to NTEP and WHO guidelines, one CDST laboratory is required for every 25 TB Units (ratio = 0.04). Using equation 3, we calculated the current national ratio by dividing the existing CDST laboratories (*N* = 97) by existing TB Units (*N* = 6,308), yielding 0.01538. The difference from the recommended ratio (0.04) is 0.02462, indicating a 61.56% national shortfall in CDST laboratory capacity (Fig. 2E).

### Availability of TB essential diagnostics as per ICMR NEDL recommendations

According to the ICMR NEDL 2019, diagnostic tests for TB differ across healthcare tiers. The detailed availability of these diagnostics in the surveyed facilities of Assam, Manipur, Tripura, HP, UP, Rajasthan, Haryana, and Delhi is presented in the sections below. The results are based on a cross-sectional survey conducted at a single point in time via in-person visits to the facilities.

### Availability of ED for TB diagnosis across seven states and one UT

**Fig 3:**
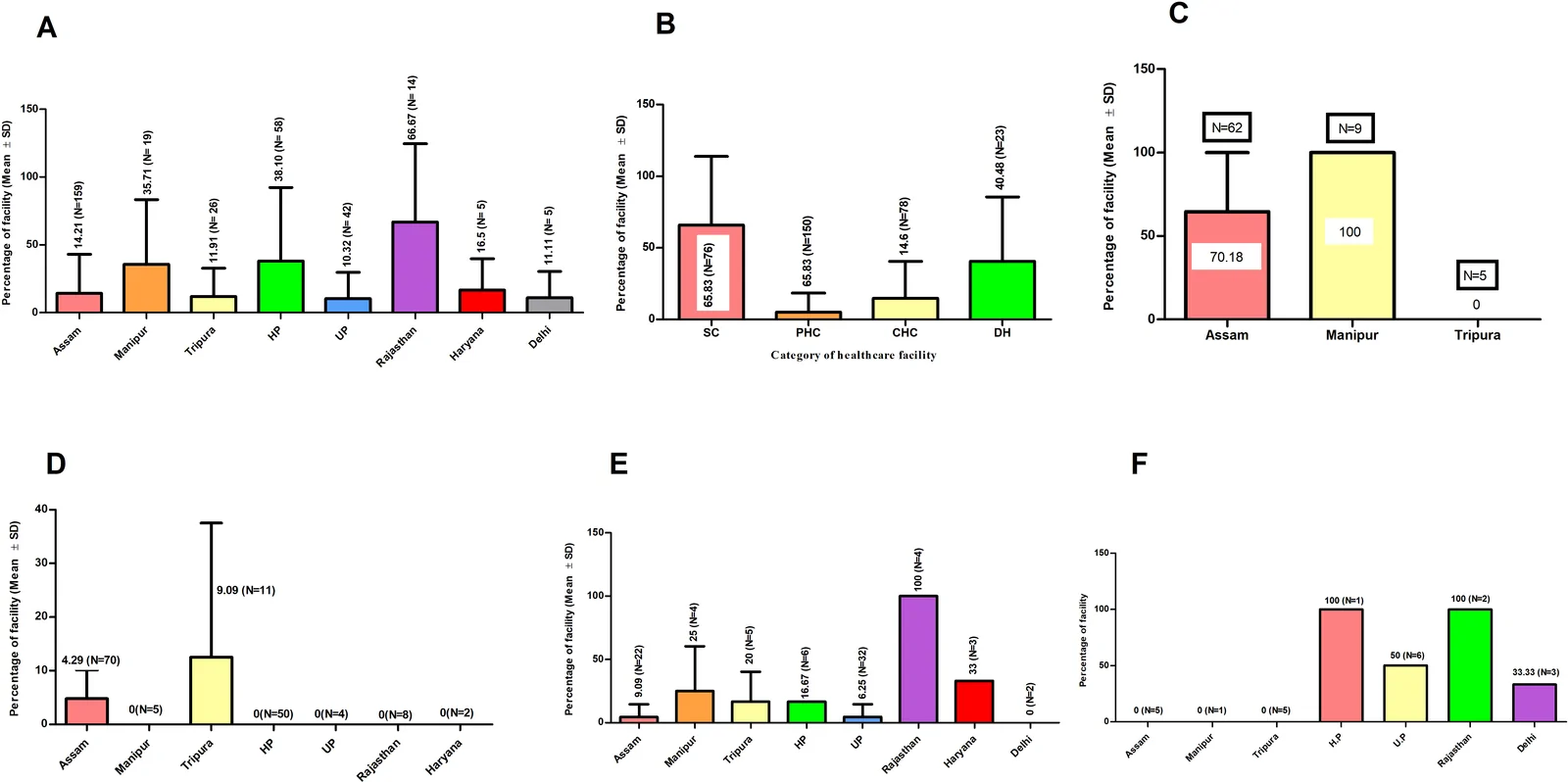
Availability of TB-ED across states and categories of healthcare facilities. (**A**) Percentage of facilities with complete TB-ED across states: mean ± SD percentage of facilities (SCs, PHCs, CHCs, and DHs) having all NEDL-2019-recommended TB diagnostic tests for their respective category. ED availability was recorded only when all required tests were present. *N* indicates the number of facilities surveyed. A chi-square test showed a significant difference in ED availability between states (*P* = 0.01; *χ*² = 4.16; df = 7). (**B**) Percentage of facilities with complete TB-ED across healthcare facility categories: mean ± SD values were derived by aggregating state-level data across facility types. ED availability was considered only when all required tests were present. *N* indicates the number of facilities surveyed. A Kruskal–Wallis test showed a significant difference across facility categories (*P* = 0.001; *χ*² = 9.01; df = 3). (**C**) Percentage of SCs with TB-ED across states: for SCs, sputum collection is the only ED for TB diagnosis (Table S2). Data are presented as the mean ± SD of the percentage of SCs with sputum collection facilities, with district-level values treated as replicates. No statistical analysis was performed due to lack of replicates in Tripura, where only one district was surveyed. *N* indicates the number of SCs surveyed. (**D**) Percentage of PHCs with TB-ED across states: two tests—AFB smear microscopy and X-ray—are included as TB-related essential diagnostics at PHCs (Table S2). PHC-level ED availability was considered only when both tests were present. District-level values were used as replicates where applicable and expressed as mean ± SD. No statistical comparison was performed due to single-district sampling in Himachal Pradesh (Sirmaur), Uttar Pradesh (Gautam Buddh Nagar), Rajasthan (Jaipur), and Haryana (Faridabad), resulting in no replicates. (**E**) Percentage of CHCs with TB-ED across states and Delhi: three tests—AFB smear microscopy, X-ray, and CBNAAT—are included as ED at CHCs. Availability is reported only when all three tests are present. State-wise values are expressed as mean ± SD, with district-level data used as replicates. No statistical analysis was performed due to limited replicates in some states/UTs. (**F**) Percentage of DHs with TB-ED across states and Delhi: four tests—AFB smear microscopy, Gram staining of pus cells by fluorescence microscopy, X-ray, and CBNAAT—are included as ED at district hospitals. Availability is reported only when all four tests are present. Values are presented as percentages of DHs with complete ED availability. No statistical analysis was performed because each district typically has a single DH, so there were no replicates.

Our survey found that complete ED availability for TB across all healthcare categories ranged from 10.32% to 66.67% across seven states and Delhi. Rajasthan showed the highest availability, with 66.67% of its 14 surveyed facilities meeting all ED requirements, while Uttar Pradesh had the lowest at 10.32% of 42 facilities. Assam, Tripura, Haryana, and Delhi also reported low coverage, with 14.21% (*N* = 159), 11.91% (*N* = 26), 16.5% (*N* = 5), and 11.11% (*N* = 5) of facilities, respectively, having the full ED set. Here, we performed the Kruskal-Wallis test, which indicated no significant difference (*P* value = 0.761) between states, suggesting similar implementation of diagnostic services across regions (Fig. 3A). At the district level, East Imphal (*N* = 9), Aligarh (*N* = 12), Budaun (*N* = 10), Jaipur (*N* = 14), and New Delhi (*N* = 1) had ≥50% of surveyed facilities equipped with complete EDs. Most other districts showed very low availability, and some reported none at all—including Hailakandi (*N* = 5) and Kamrup Metro (*N* = 3) in Assam; Unakoti (*N* = 5) in Tripura; Bulandshahr (*N* = 4) and Kasganj (*N* = 4) in UP; and single surveyed facilities in four districts of Delhi (Fig. S2A). Statistical comparisons were not performed at the district level for reasons outlined in the Materials and Methods. Overall, across all 327 surveyed facilities, SCs showed the highest availability (65.83%), while PHCs had the lowest (4.94%). CHCs demonstrated 14.6% availability, and DHs showed 40.48% compliance. Differences across facility categories were statistically significant (*P* value = 0.029) (Fig. 3B).

### Availability of ED for TB diagnosis across different regions and categories of healthcare facilities

We surveyed ED availability for TB across 76 SCs in Assam (*N* = 62), Manipur (*N* = 9), and Tripura (*N* = 5). Since sputum collection is the only SC-level ED under ICMR NEDL-2019, our analysis focused on this service. All SCs in Manipur had sputum collection, compared with 70% in Assam and 0% in Tripura (Fig. 3C). At the district level, Sribhumi and both Imphal districts showed 100% availability, whereas Cachar had 29.17%, and South Tripura had none (Fig. S2B). At the PHC level (*N* = 150), ED availability was defined by the presence of AFB microscopy and mobile or standard X-ray. ED coverage was consistently poor, ranging from 0% to 10%. No PHCs in Manipur, Himachal Pradesh, Uttar Pradesh, Rajasthan, or Haryana had the required diagnostics. Limited availability was observed in Assam (4.29%) and Tripura (9.9%) (Fig. 3D). District-level variation is shown in Fig. S2C. At the CHC level (*N* = 78), EDs include AFB microscopy, X-ray, and CBNAAT. Overall availability remained low (0%–35%). Coverage was 9.09% in Assam, 25% in Manipur, 20% in Tripura, 16.67% in Himachal Pradesh, 6.25% in Uttar Pradesh, 33% in Haryana, and 0% in Delhi. Rajasthan was the only state where all four surveyed CHCs had complete EDs (Fig. 2E). District-level availability ranged from 100% in Jaipur to 0% in 12 districts (Fig. S2D). At the DH level, the four required EDs under NEDL-2019 include AFB microscopy, fluorescence microscopy for pus cells, X-ray, and CBNAAT. Complete ED availability was not observed in any of the surveyed DHs in Assam (*N* = 5), Manipur (*N* = 1), and Tripura (*N* = 5). In contrast, one DH in Himachal Pradesh, two in Rajasthan, and one of three in Delhi met all requirements (Fig. 2F). The statistical analysis of the data sets in Fig. 2C through F and the district-level percentage availability of ED (Fig. S2 through D) could not be performed for reasons detailed in the Materials and Methods.

### Availability of each ED test for TB diagnosis across different regions and categories of healthcare facilities

**Fig 4:**
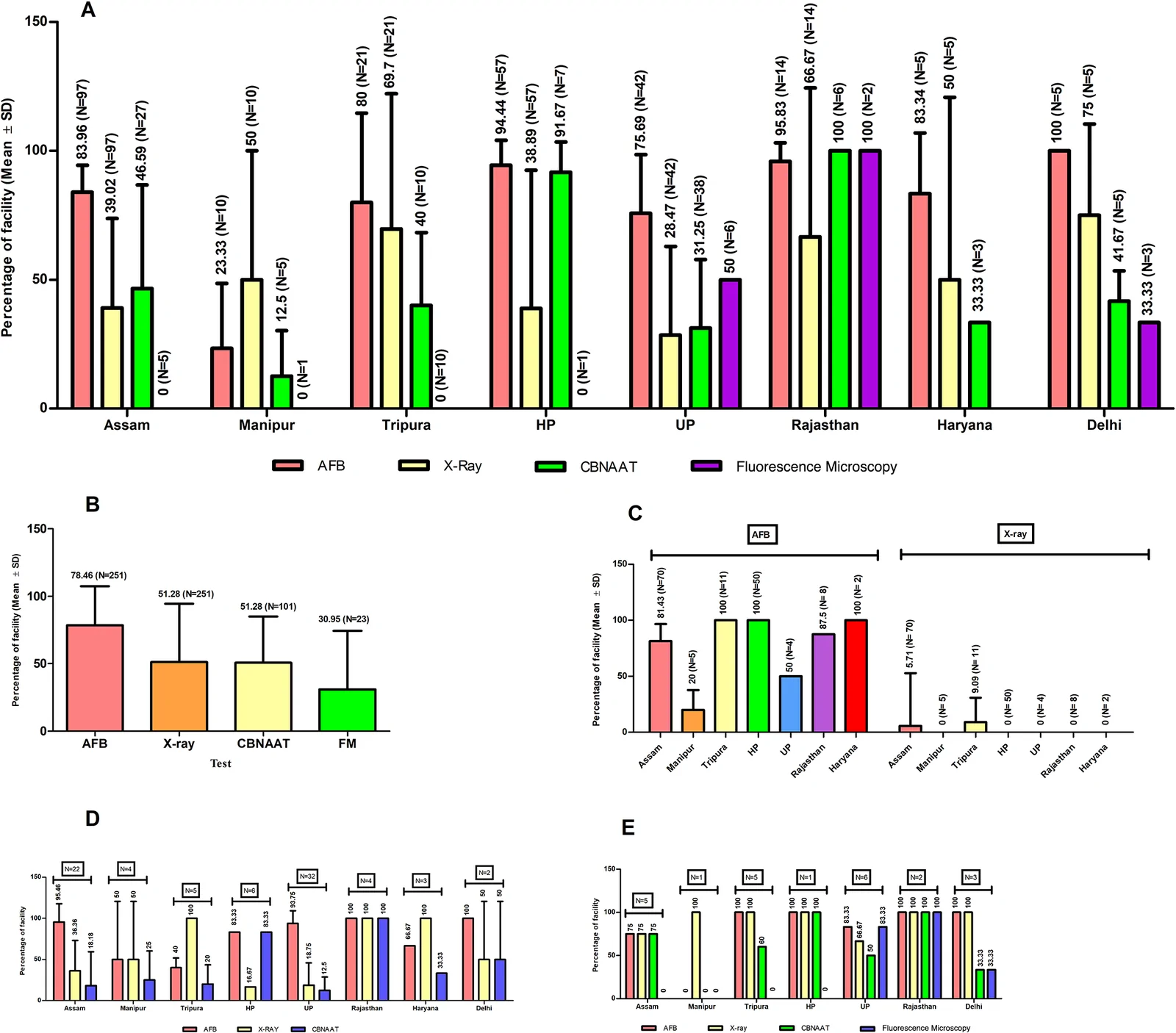
Availability of individual ED tests for TB diagnosis across states/UT and categories of healthcare facilities. (**A**) Test-wise availability across states and the Union Territory of Delhi: four ED tests—AFB smear microscopy, fluorescence microscopy of pus cells, X-ray, and CBNAAT—were assessed across all facility types. This panel shows the cumulative percentage of facilities having each test in each state/UT. Data are expressed as mean ± SD; *N* indicates the number of facilities surveyed. Differences in test-wise availability across regions were statistically significant (Kruskal–Wallis test; *P* = 0.035; *χ*² = 8.63; df = 3). (**B**) Overall availability of each ED test across all states and facility types: data represent the percentage of facilities having each test, aggregated across all states and healthcare categories. Results are presented as mean ± SD; *N* indicates the number of facilities surveyed. Significant differences were observed in overall test availability (Kruskal–Wallis test; *P* = 0.029; *χ*² = 9.06; df = 3). (**C**) Availability of AFB smear microscopy and X-ray in PHCs across states: district-level values were used as replicates where applicable and expressed as mean ± SD. No statistical analysis was performed due to single-district sampling in Himachal Pradesh (Sirmaur), Uttar Pradesh (Gautam Buddh Nagar), Rajasthan (Jaipur), and Haryana (Faridabad), resulting in no replicates for comparison. (**D**) Availability of AFB smear microscopy, X-ray, and CBNAAT in CHCs across states and Delhi: district-level percentages were used as replicates where available and presented as mean ± SD. No statistical analysis was performed due to single-district sampling in Himachal Pradesh (Sirmaur), Rajasthan (Jaipur), and Haryana (Faridabad), which limited the number of replicates for comparison. (**E**) Availability of ED tests in DHs across states and Delhi: four tests—AFB smear microscopy, fluorescence microscopy, X-ray, and CBNAAT—were assessed. Data are presented as the percentage of DHs with each test; *N* indicates the number of DHs surveyed. No statistical analysis was performed because most districts have only one DH, resulting in no replicates.

The AFB smear test, an ED for PHCs, CHCs, and DHs, was the most widely available compared to X-ray, CBNAAT, and FM. Its lowest availability was in Manipur (23.33%, *N* = 10), while in the other six states and Delhi, 75%–100% of facilities had it (Fig. 4A). X-ray availability was also poor across all states, with none reaching 50% operational coverage; Uttar Pradesh showed the lowest availability (28.47%, *N* = 42). CBNAAT, recommended for CHCs and DHs, was also limited, except in Himachal Pradesh (91.67%, *N* = 7) and Rajasthan (100%, *N* = 6). FM for pus-cell detection, recommended only for DHs, showed highly variable availability: no DHs in Assam, Tripura, or Manipur had the test; Rajasthan (100%, *N* = 2) and Uttar Pradesh (50%, *N* = 6) had the highest availability. Delhi showed a moderate presence (33.33%, *N* = 3). Resource constraints limited the number of DHs surveyed (*N* = 23). The Kruskal–Wallis test indicated a statistically significant difference (*P* value = 0.035) among the tests across regions.

Overall, after assessing the percentage availability of facilities offering each diagnostic test—aggregated across categories of health-care facilities and regions—we found that AFB microscopy was available in 78.46% of recommended facilities, X-ray services in 51.28% of 251 PHCs/CHCs/DHs, CBNAAT in 51.28% of 101 CHCs/DHs, and FM in only 30.95% of 23 DHs. The differences in availability among these tests were statistically significant (*P* value = 0.029), as indicated by the Kruskal–Wallis test (Fig. 4B).

In the health care category-wise comparison of tests, SCs were excluded because NEDL-2019 recommends only sputum collection as the ED for TB at this level (Fig. 1), which does not fall under any test. In PHCs, AFB availability ranged from 50% to 100% across states except Manipur (20%, *N* = 5), whereas X-ray availability was uniformly low (0%–10%) (Fig. 4C). Formal statistical testing was not feasible owing to the limited number of district-level replicates for PHC-wise comparison, as outlined in the Materials and Methods.

District-wise PHC data are shown in Fig. S3A, and the difference was significant (*P* value = 0.0001) by the Mann-Whitney *U* test. Across CHCs, most states and Delhi had 80%–100% AFB availability, except Manipur (50%, *N* = 4). X-ray availability varied widely and was particularly low in Uttar Pradesh (18.75%, *N* = 32) and Himachal Pradesh (16.67%, *N* = 6). CBNAAT availability was highest in Himachal Pradesh (83.33%, *N* = 6), Delhi (50%, *N* = 2), and Rajasthan (100%, *N* = 4), while the remaining states had only 10%–40% coverage (Fig. 4D). No statistical analysis could be performed due to the limited number of district-level replicates available for CHC-wise comparison, as described in the Materials and Methods.

District-wise CHC details are provided in Fig. S3B, and the Kruskal-Wallis test showed significant differences (*P* value = 0.001) among them. At the DH level, the availability of AFB, X-ray, and CBNAAT was generally highest across states and Delhi, except in Manipur, where only AFB was available. FM availability remained low in Assam, Tripura, Manipur, and Delhi, but was highest in Rajasthan (100%, *N* = 2) and Uttar Pradesh (83.33%, *N* = 6) (Fig. 4E). Statistical analysis was not performed due to limitations described in the Materials and Methods.

### Regional and facility-level availability of essential diagnostics related to CBC and CMP panels

**Fig 5:**
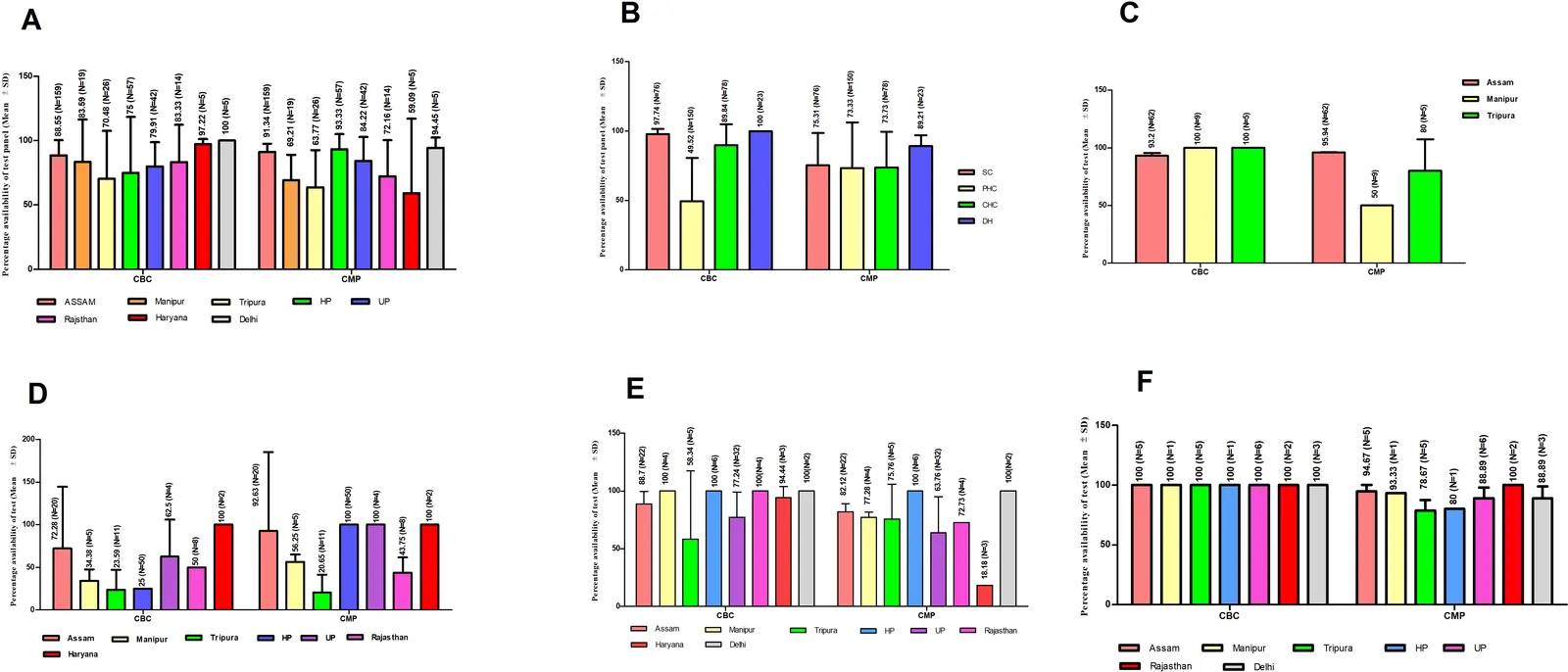
Availability of CBC and CMP test panels across states and categories of healthcare facilities. (**A**) Availability across states and the Union Territory of Delhi: the figure shows the percentage availability of CBC and CMP test panels, as recommended in NEDL-2019 (Fig. 1), across all categories of healthcare facilities in the selected states and UT of Delhi. The percentage of available tests was calculated separately for CBC and CMP as a proportion of the total recommended tests. Data are presented as mean ± SD; *N* indicates the number of facilities surveyed. An independent samples *t*-test showed no significant difference in availability across states (*P* = 0.322; *t* = 1.026; df = 14). (**B**) Availability across healthcare facility categories: data represent the percentage availability of CBC and CMP test panels across facility types, aggregated from state-level values. Results are presented as mean ± SD; *N* indicates the number of facilities surveyed. No significant difference was observed across categories (Mann–Whitney *U* test; *P* = 0.248; *U* = 4.0). (**C**) Availability in SCs across three states: data from Assam, Manipur, and Tripura are shown. Percentage availability was calculated separately for CBC and CMP, expressed as a proportion of the recommended tests. Results are presented as mean ± SD; *N* indicates the number of facilities surveyed. No significant difference was observed among states (Mann–Whitney *U* test; *P* = 0.121; *U* = 1.0). (**D**) Availability in PHCs across seven states: data from Assam, Manipur, Tripura, Himachal Pradesh, Uttar Pradesh, Rajasthan, and Haryana are shown. Results are expressed as mean ± SD; *N* indicates the number of facilities surveyed. No significant difference was observed across states (independent samples *t*-test; *P* = 0.226; *t* = −1.28; df = 12). (**E**) Availability in CHCs across seven states and the UT of Delhi: data include Assam, Manipur, Tripura, Himachal Pradesh, Uttar Pradesh, Rajasthan, Haryana, and Delhi. Results are presented as mean ± SD; *N* indicates the number of facilities surveyed. No significant difference was observed across regions (independent samples *t*-test; *P* = 0.149; *t* = 1.526; df = 14). (**F**) Availability in DHs across six states and the UT of Delhi: data include Assam, Manipur, Tripura, Himachal Pradesh, Uttar Pradesh, Rajasthan, and Delhi. Results are presented as mean ± SD; *N* indicates the number of facilities surveyed. A significant difference was observed in availability across regions (Mann–Whitney *U* test; *P* = 0.004; *U* = 7.0).

The CBC and CMP test panels are essential for TB treatment and management. We assessed their availability across 327 healthcare facilities in seven states and one union territory. The CBC panel included six tests (RBC, WBC/TLC, platelets, hemoglobin, hematocrit, and mean corpuscular volume test), and the CMP panel included 15 tests, as recommended under ICMR–NEDL 2019 (Fig. 1). Based on these guidelines, we calculated the percentage availability of required tests in each facility. Across states and Delhi, CBC availability ranged from 70% to 100%, with Tripura having the lowest (70.48%, *N* = 26). CMP availability showed a similar pattern (59%–95%), with Haryana lowest (59.09%, *N* = 5). For statistical testing, we used an independent-samples *t*-test, which showed no significant difference (*P* value = 0.322) between the CBC and CMP test panels across states (Fig. 5A).

By facility type, CBC availability was high in SCs, CHCs, and DHs (89%–100%) but much lower in PHCs (49.52%, *N* = 150). CMP availability was 73%–76% in SCs, PHCs, and CHCs, and highest in DHs (89.21%, *N* = 23), with statistically non-significant differences (*P* value = 0.248) between CBC and CMP test panels across various centers of healthcare, as indicated by the Mann-Whitney *U* test (Fig. 5B).

State-level CBC availability in SCs from Assam, Manipur, and Tripura was 93%–100%, while CMP availability was lowest in Manipur (50%, *N* = 9). Also, from the Mann-Whitney *U* test, we find no significant difference (*P* value = 0.121) between the CBC and CMP test panels in the SCs of these three states (Fig. 5C). At the district level, CBC availability was lowest in West and East Imphal (both 50%) (Fig. S4A). The Mann-Whitney *U* test showed no significant differences (*P* value = 0.071) in CBC and CMP test panels among SCs across states or districts.

Among PHCs, CBC availability was poor in HP (25%), Tripura (23.59%), and Manipur (34.38%), although other states showed better performance. CMP availability at PHCs was generally higher than CBC, except in Tripura (20.65%) (Fig. 5D). From the independent-samples *t*-test, we observe no significant difference (*P* value = 0.226) in the availability of CBC and CMP test panels across PHCs in the states. The Mann-Whitney *U* test showed a statistically significant difference (*P* value = 0.010) between CBC and CMP test panels among PHCs at the district level (Fig. S4B).

In CHCs, CBC availability was lowest in Tripura (58.34%, *N* = 5) and 75%–100% elsewhere. CMP availability was 70%–100% across most states, but very low in Haryana (18.18%, *N* = 3) (Fig. 5E). District-level CMP availability was particularly low in West Tripura, Budaun, and Faridabad (Fig. S4C). An independent-samples *t*-test showed no significant difference (*P* value = 0.149) in the availability of CBC and CMP test panels across states. In contrast, the Mann-Whitney *U* test showed a significant difference (*P* value = 0.010) between the CBC and CMP test panels across districts.

At the DH level, CBC availability was 100% in all states and Delhi. CMP availability was also high, except for slightly lower values in Tripura (78.67%, *N* = 5) and Uttar Pradesh (88.89%, *N* = 6) (Fig. 5F). There is a significant variation (*P* value = 0.004) in the availability of CBC and CMP test panels across states at the DH level, as indicated by the Mann-Whitney *U* test.

## DISCUSSION

The goal of eliminating TB remains challenging, as recent reports indicate that TB continues to be the leading infectious disease worldwide (31). India alone accounts for approximately one-fourth of the global TB burden, despite sustained efforts by the Government of India toward TB elimination (2). The experience of the COVID-19 pandemic has highlighted the critical importance of early detection and isolation in controlling infectious diseases (32). India’s growing population, demographics, and geographical complexities make it extremely challenging to implement the TB elimination program equally across the entire population of India. For example, TB incidence among tribal communities has been reported at 703 per 100,000 population—more than threefold higher than the national estimate of 199 per 100,000 (33). Such uneven distribution of TB incidence suggests gaps in the current TB management system. The persistence of these challenges indicates structural gaps in health system capacity, particularly at the primary and secondary levels where initial diagnosis and treatment initiation occur (34). This study evaluated the existing TB control strategies to identify systemic weaknesses and improve the effectiveness of TB elimination efforts.

Within the organizational architecture of tuberculosis control in India, TB Units constitute the first operational and administrative tier. TB Units are responsible for diagnosing, treating, and tracking TB cases within defined geographical areas. Complementing these units are CDST laboratories, which provide specialized diagnostic services to identify drug resistance and guide complex treatment regimens. The TB Units typically collect samples from presumptive TB patients and refer them to CDST laboratories for confirmation and resistance testing. Thus, the TB Units and CDST laboratories together form the backbone of India’s diagnostic referral system and play a pivotal role in ensuring timely and accurate diagnosis and the flow of relevant data to the NTEP (35). Our findings reveal substantial disparities. The uneven distribution of these critical tuberculosis management infrastructures suggests a concentration of facilities in certain urban and semi-urban locations, leaving rural, remote, and socio-economically disadvantaged regions underserved.

As part of the NTEP, the TB Units often function within or in coordination with PHCs or CHCs. Therefore, understanding the diagnostic capacity of these facilities is central to evaluating the broader impact on TB control. India operates a three-tier public health system: primary care, secondary care, and tertiary care (specialist and teaching hospitals). Each tier plays a distinct role in service delivery (Fig. 1). Primary-level facilities serve as the first point of contact for rural populations; secondary facilities provide more advanced diagnostic and treatment services; and tertiary institutions manage complex and severe cases. Additionally, each tier of the public healthcare system is assigned a normative population coverage (sub-center: 3,000–5,000; primary health center: 20,000–30,000; community health center: 80,000–120,000; district hospital: ~2.5 million) (Fig. 1). These benchmarks can serve as proxy denominators for estimating the population that may be underserved when essential diagnostic services are unavailable at a given facility. The survey sites were based on our logistics and current collaborations in the regions. This limitation, due to fewer states being included in data collection, may introduce potential selection bias, but these sites reflect TB diagnostic facilities nationwide. They cover remote North-Eastern states, high-population northern states, and an urban metropolitan area (Delhi). This captures the geographic diversity and reflects the healthcare infrastructure and access to diagnostic services. Each healthcare facility was assessed at a single time point; the 18-month survey period may have introduced temporal heterogeneity, as changes in diagnostic infrastructure, resource availability, or health system practices could have occurred during the data collection period, potentially affecting comparability across surveyed sites.

Our assessment of essential diagnostics for TB across multiple states revealed that most primary healthcare settings rely primarily on AFB smear microscopy, a relatively less sensitive diagnostic method. This reliance increases the likelihood of missed or delayed diagnosis. In addition, chest X-ray facilities are rarely available at PHCs, further contributing to the under-detection of TB cases. We also observed that only a small proportion of PHCs and CHCs meet the recommended standards for the complete availability of essential TB diagnostics. Molecular diagnostic methods, such as CBNAAT, which the WHO recommends for testing all presumptive TB cases and for early diagnosis of drug resistance, remain underutilized. It was reported that only about 33% of presumptive cases are tested using CBNAAT (19). Our findings suggest that CBNAAT facilities are not uniformly distributed even within CHCs, with a majority lacking access to this technology. Given standard population coverage, the absence of TB diagnostics at a PHC facility may affect 30,000–50,000 individuals, while the lack of CBNAAT and other essential diagnostics at a CHC may deprive approximately 80,000–120,000 people of appropriate diagnostic services. These findings, consistent with earlier reports, highlight the need for equitable implementation of molecular diagnostic facilities across all CHCs and, where feasible, at the PHC level, as they are typically the first point of contact for patients seeking healthcare.

Although about 50% of TB patients first seek care in the private sector, this group is eventually enrolled in the National TB Elimination Program under the Directly Observed Treatment Schedule. Moreover, for most of the Indian population seeking initial care in the private sector, access is often limited to those who can afford it. There are about 2,000 private laboratories in India accredited by the National Accreditation Board for Testing and Calibration Laboratories, but TB diagnostic services in the private sector are still limited (36). While there are 84 NTEP-certified CDST laboratories (30) in the public sector, only 13 are in the private sector. The study thus accounted for private-sector infrastructure capable of TB drug sensitivity testing. Moreover, TB diagnostic and treatment services—particularly CDST—remain largely concentrated within the public healthcare system due to stringent biosafety requirements and the need for standardized containment measures. More importantly, CDST and TB are strictly maintained and monitored by the government under the NTEP (37).

In addition to the availability of TB diagnostic and drug sensitivity tools to assess treatment monitoring for TB, the availability of basic metabolic and hematological tests, including CBC and CMP, in public healthcare settings is also required. These parameters are essential for monitoring patient response to therapy and detecting adverse drug reactions (38, 39). This analysis is particularly relevant because TB Units, which are primarily responsible for treatment delivery and monitoring, are typically linked to PHCs and CHCs. Therefore, evaluating the availability of essential CBC- and CMP-related diagnostics provides important insight into the functional capacity of the TB management system at the peripheral level. The availability of CBC and CMP diagnostics was relatively better than that of TB-specific diagnostics; however, significant gaps persist in public healthcare facilities, particularly for the Comprehensive Metabolic Panel at the Community Health Center level. This indicates an unequal distribution of treatment monitoring services across PHCs and CHCs, irrespective of geographic region. As a result, these facilities often depend heavily on DHs for routine monitoring, which overburdens higher-level centers and increases the risk of delays, errors, and misdiagnosis. Achieving TB elimination in India will require optimizing primary health care infrastructure, particularly by systematically scaling up NAAT-based diagnostics for early detection and improving the availability of CBC and CMP testing for treatment monitoring and preventing adverse drug reaction-related morbidity. In a previous review article, we also optimized and outlined a range of diagnostic recommendations for TB diagnosis derived from international agency guidelines and underscored the importance of revising national diagnostic policies to align with country-specific needs (40). The NAATs have substantially improved rapid detection of *Mycobacterium tuberculosis*; their ability to comprehensively detect emerging multidrug-resistant and extensively drug-resistant strains remains constrained by target-limited molecular design and evolving resistance mechanisms. On the other hand, the next-generation sequencing technologies offer expanded resistance profiling, but challenges related to cost, infrastructure, bioinformatics capacity, and scalability limit widespread implementation, particularly in resource-constrained settings.

### CONCLUSIONS

This study provides one of the first multi-state assessments of the availability of tuberculosis-related essential diagnostic tests in public healthcare facilities across diverse regions of India. The findings reveal substantial gaps in the implementation of NEDL recommendations, with marked disparities across healthcare tiers and states. Primary Health Centers demonstrated the lowest diagnostic readiness, while the availability of key TB screening and diagnostic services, including chest radiography, cartridge-based point-of-care molecular nucleic acid testing, and culture-based testing, remained suboptimal in many facilities. Our national gap analyses of TB elimination program infrastructures identified significant shortfalls in both Tuberculosis Treatment Units and associated CDST laboratories relative to population-based requirements as prescribed by the National TB Elimination Program, India, and the World Health Organization. While AFB smear microscopy and chest radiography continue to play important roles in TB screening, reliance on these modalities alone is insufficient to achieve India’s TB elimination targets. Expansion of rapid molecular diagnostics, coupled with the strengthening of the timely detection of drug-resistant tuberculosis and effective treatment and management. The observed diagnostic gaps highlight the need for targeted investments in laboratory infrastructure, workforce capacity, and peripheral healthcare. Collectively, these findings provide important evidence for policymakers and TB elimination program managers, emphasizing that successful tuberculosis elimination requires a balanced and integrated CDST laboratory network that combines rapid molecular technologies with robust culture-based surveillance systems. Addressing these systemic gaps will be critical for strengthening India’s TB control efforts and achieving the goals of the National TB Elimination Program. The functional CDST Laboratory network will also help elucidate the dynamic biological mechanisms underlying the evolution of Mycobacterial drug resistance, an emerging public health threat in India.

## Supplementary Material

Reviewer comments

## Data Availability

All publicly available data analyzed in this manuscript are available as described and referred to in the Materials and Methods section "Collection of data regarding the distribution of TB Units across India" and "Assessment of the distribution of CDST-based TB diagnostics across India." The data analyzed are presented in the supplementary material (Table S1).

## AFTER EPUB

*[This article was published on 11 August 2026 with errors in the equal contribution footnote and Fig. 4. The errors were corrected in the current version, posted on 18 August 2026.]*

## SUPPLEMENTAL MATERIAL

Fig S1 spectrum.00703-26-s0001.jpg Legend Available in Supplementary File R1.

Fig S2 spectrum.00703-26-s0002.jpg Legend Available in Supplementary File R1.

Fig S3 spectrum.00703-26-s0003.jpg Legend Available in Supplementary File R1.

Fig S4 spectrum.00703-26-s0004.jpg Legend Available in Supplementary File R1.

Fig S1 to S4 and Tables S1 S2 spectrum.00703-26-s0005.docx Supplementary Figures and Tables. All legends are in the file.

OPEN PEER REVIEW reviewer-comments.pdf An accounting of the reviewer comments and feedback.

ASM does not own the copyrights to Supplemental Material that may be linked to, or accessed through, an article. The authors have granted ASM a non-exclusive, world-wide license to publish the Supplemental Material files. Please contact the corresponding author directly for reuse.
