# Supplementary material for: Evaluating the nationwide availability of essential diagnostics for tuberculosis toward achieving a TB-free India
Source: Microbiol Spectr. 2026 Aug 11;14(9):e00703-26. doi: 10.1128/spectrum.00703-26 (PMC13532310; doi:10.1128/spectrum.00703-26)
Supplement: Supplementary file 1 [file reviewer-comments.pdf]

AMERICAN SOCIETY FOR MICROBIOLOGY | Microbiology Spectrum

Evaluating the nationwide availability of essential diagnostics for tuberculosis towards achieving a TB-free India

Mustafa Barbhuiya, Biswajit Chakraborty, Amaj Laskar, Vipin Kumar, Rinki Kumar, Kajal Arora, Zeeshan Mustafa, Anjam Barbhuiya, Afnan Hussain, Ashrafun Parvin, Leichombam Singh, Kanu Shil, Sumit Roy, Mohammad Khan, and Anamika Baruah

Corresponding Author(s): Mustafa Barbhuiya, Baystate Medical Center

Review Timeline:

Submission Date:	February 27, 2026
Editorial Decision:	April 6, 2026
Revision Received:	May 27, 2026
Editorial Decision:	June 18, 2026
Revision Received:	June 21, 2026
Accepted:	June 27, 2026

Editor: Sophia Georghiou

Reviewer(s): The reviewers have opted to remain anonymous.

Transaction Report:

(Note: With the exception of the correction of typographical or spelling errors that could be a source of ambiguity, letters and reports are not edited. The original formatting of letters and referee reports may not be reflected in this compilation.)

DOI: https://doi.org/10.1128/spectrum.00703-26

Re: Spectrum00703-26 (Evaluating the nationwide availability of essential diagnostics for tuberculosis towards achieving a TB-free India)

Dear Dr. Mustafa A Barbhuiya:

Thank you for the privilege of reviewing your work. Below you will find my comments, instructions from the Spectrum editorial office, and the reviewer comments.

Please return the manuscript within 60 days; if you cannot complete the modification within this time period, please contact me. If you do not wish to modify the manuscript and prefer to submit it to another journal, notify me immediately so that the manuscript may be formally withdrawn from consideration by Spectrum.

Revision Guidelines
To submit your modified manuscript, log into the submission site at https://spectrum.msubmit.net/cgi-bin/main.plex. Go to Author Tasks and click the appropriate manuscript title to begin. The information you entered when you first submitted the paper will be displayed; update this as necessary. Note the following requirements:

• Upload point-by-point responses to the issues raised by the reviewers in a file named "Response to Reviewers," NOT in your cover letter.
• Upload a compare copy of the manuscript (without figures) as a "Marked-Up Manuscript" file.
• Upload a clean .DOC/.DOCX version of the revised manuscript and remove the previous version.
• Each figure must be uploaded as a separate, editable, high-resolution file (TIFF or EPS preferred), and any multipanel figures must be assembled into one file.
• Any supplemental material intended for posting by ASM should be uploaded with their legends separate from the main manuscript. You can combine all supplemental material into one file (preferred) or split it into a maximum of 10 files with all associated legends included.

For complete guidelines on revision requirements, see our Submission and Review Process webpage. Submission of a paper that does not conform to guidelines may delay acceptance of your manuscript.

Data availability: ASM policy requires that data be available to the public upon online posting of the article, so please verify all links to sequence records, if present, and make sure that each number retrieves the full record of the data. If a new accession number is not linked or a link is broken, provide Spectrum production staff with the correct URL for the record. If the accession numbers for new data are not publicly accessible before the expected online posting of the article, publication may be delayed; please contact production staff (Spectrum@asmusa.org) immediately with the expected release date.

Publication Fees: For information on publication fees and which article types are subject to charges, visit ourwebsite. If your manuscript is accepted for publication and any fees apply, you will be contacted separately about payment during the production process; please follow the instructions in that e-mail. Arrangements for payment must be made before your article is published.

ASM Membership: Corresponding authors may join or renew ASM membership to obtain discounts on publication fees. Need to upgrade your membership level? Please contact Customer Service at Service@asmusa.org.

The ASM Journals program strives for constant improvement in our submission and publication process. Please tell us how we can improve your experience by taking this quick Author Survey.

Thank you for submitting your paper to Spectrum.

Sincerely,
Sophia Georghiou
Editor
Microbiology Spectrum

Reviewer #1 (Comments for the Author):

This study addresses an important and timely topic-TB diagnostic infrastructure gaps in India. The combination of national-level data extraction with field-level surveys provides valuable insights. However, several methodological, analytical, and presentation issues require attention.

1. The convenience sampling approach ("surveys were conducted only in locations where logistical support was available") introduces significant selection bias. The authors should explicitly acknowledge this as a limitation and discuss directionality of

bias-facilities in accessible areas likely have better infrastructure than truly remote regions, suggesting the gaps reported may be underestimates.

2.The highly uneven sampling across states (e.g., 159 facilities in Assam vs. 5 in Haryana; 5 districts in some states vs. 1 in others) limits meaningful inter-state comparisons. Consider removing state-level comparative statistics or reframing as exploratory/descriptive rather than comparative.

3. Justification for state selection is absent. Why these seven states and Delhi? Do they represent geographic, demographic, or epidemiologic diversity? A rationale should be provided.

4. The 18-month survey window (November 2023-April 2025) is problematic. Policy changes, equipment procurement, or infrastructure improvements during this period could affect comparability. Address whether any temporal adjustments were made.

5. The definition of "availability" needs clarification. Does this mean physical presence of equipment, functional equipment with reagents, or actual service provision? Prior literature demonstrates these differ substantially

6. Private sector exclusion: Approximately 50% of Indian TB patients first seek care privately. The CDST gap calculations using only public facilities may significantly overestimate true gaps. Discuss this limitation explicitly.

7.Human resources: Equipment without trained personnel is non-functional. Staffing data (laboratory technicians, radiographers) should be collected or acknowledged as a limitation.

8. Utilization data: Availability ≠ utilization. Were any data collected on actual test volumes or patient access?

9.The abstract states "nationwide assessment of 327 healthcare facilities across seven states"-this is not truly "nationwide" given India has 28 states and 8 UTs. Revise language to "multi-state assessment" or similar.

10. In methods, justification for choosing parametric vs. non-parametric tests is vague. Specify normality testing methods used.

11.The discussion is lengthy and somewhat repetitive of Results. Streamline to focus on interpretation, comparison with literature, and implications.

12. Reference 2 is cited as "Global tuberculosis report 2025" but contains 2024 data-verify accuracy.

13. Several references are to government websites or portals without access dates or archived links

Reviewer #2 (Comments for the Author):

Overall Assessment This manuscript addresses an important topic-the availability of TB diagnostic modalities across health care facilities in India. The findings have value, but the manuscript requires revision to improve clarity, accessibility, and alignment with the journal's audience.

Major Comments
1. Acronyms and region specific terminology The manuscript uses many acronyms and India specific terms (e.g., ICMR, PHC, CHC, lakhs, Nikshay) without defining them. For an international readership, all acronyms and local terminology should be introduced clearly at first use.
2. Fit for the journal and special series While the work is not a microbiology study in the traditional sense, it does contribute to discussions on diagnostic access. Strengthening the link to the special series themes-point of care implementation, equitable access, and diagnostics for underserved populations-would help clarify its relevance.
3. Missing conclusion The manuscript does not include a conclusion section. A concise conclusion summarizing the key findings, their implications, and potential next steps would significantly strengthen the paper.
4. Systems level context A simple diagram outlining the tiers of the Indian health care system and the diagnostic services available at each level would greatly improve clarity for readers unfamiliar with the structure.
5. Diagnostic capacity vs. equipment presence Reporting availability of equipment is helpful, but the manuscript should also address functionality, maintenance, and availability of trained personnel, as these factors determine true diagnostic capacity.
6. Figures and supplementary materials Figures and supplementary items need clear labeling and referencing to guide the reader.
7. Writing quality The manuscript would benefit from substantial editing for grammar, structure, and readability. The scientific insights are interesting-particularly regarding availability of AFB microscopy, X ray, CBNAAT, and FM-but the writing style and

heavy use of unexplained acronyms obscure these strengths. A thorough language revision is recommended.

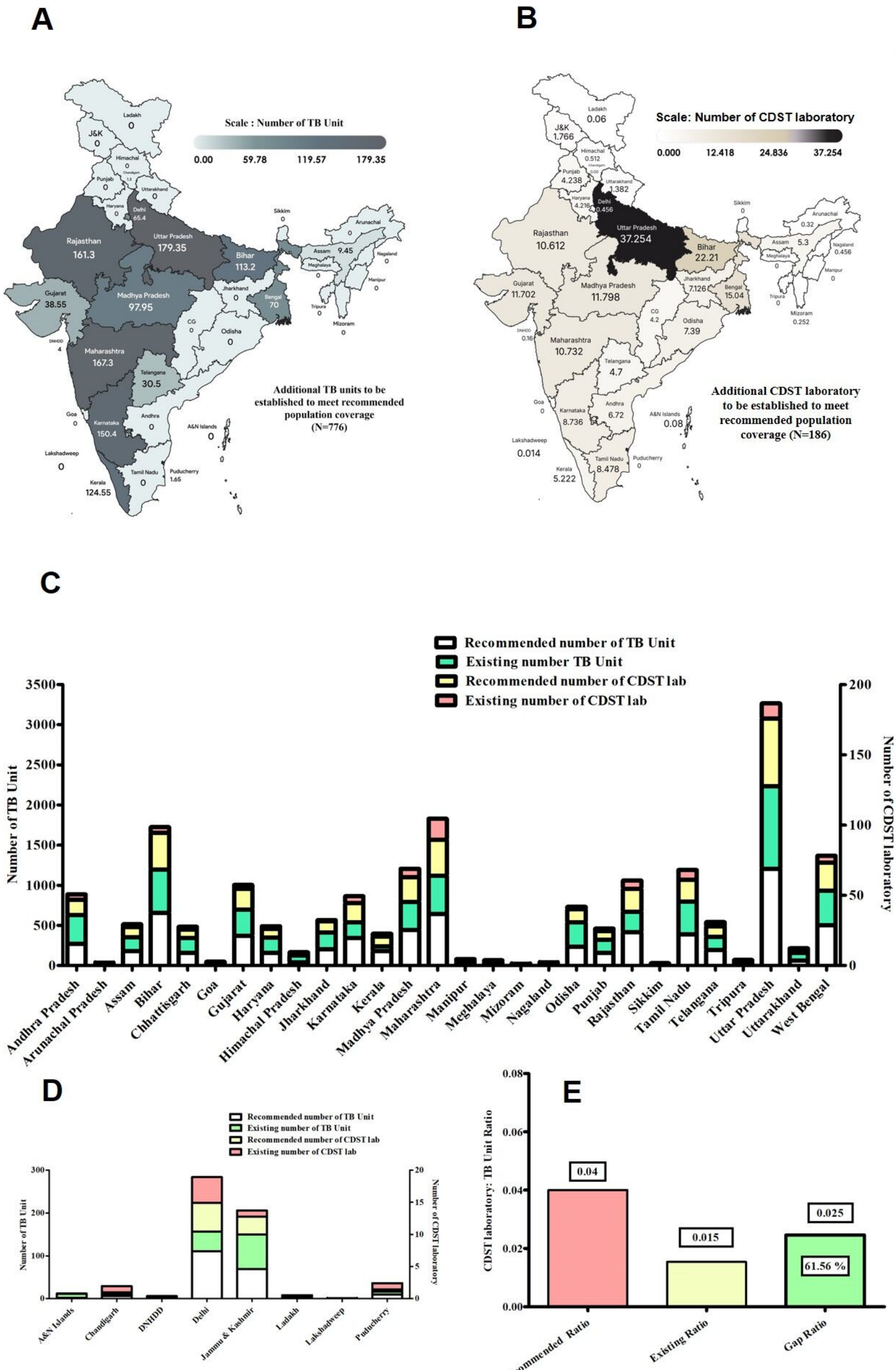

A
Scale : Number of TB Unit
0.00
59.78
119.57
179.35
Ladakh 0
J&K 0
Himachal 0
Chandigarh 1.3
Punjab 0
Uttarakhand 0
Haryana 0
Delhi 65.4
Rajasthan 161.3
Uttar Pradesh 179.35
Sikkim 0
Arunachal 0
Assam 9.45
Nagaland 0
Meghalaya 0
Manipur 0
Bihar 113.2
Gujarat 38.55
Madhya Pradesh 97.95
Jharkhand 0
Bengal 70
Tripura 0
Mizoram 0
DNHDD 4
CG 0
Odisha 0
Maharashtra 167.3
Telangana 30.5
Goa 0
Karnataka 150.4
Andhra 0
A&N Islands 0
Lakshadweep 0
Tamil Nadu 0
Puducherry 1.65
Kerala 124.55
Additional TB units to be established to meet recommended population coverage (N=776)
B
Scale: Number of CDST laboratory
0.000
12.418
24.836
37.254
Ladakh 0.06
J&K 1.766
Himachal 0.512
Chandigarh 0.00
Punjab 4.238
Uttarakhand 1.382
Haryana 4.216
Delhi 0.456
Sikkim 0
Arunachal 0.32
Rajasthan 10.612
Uttar Pradesh 37.254
Assam 5.3
Nagaland 0.456
Meghalaya 0
Bihar 22.21
Manipur 0
Gujarat 11.702
Madhya Pradesh 11.798
Jharkhand 7.126
Bengal 15.04
Tripura 0
Mizoram 0.252
DNHDD 0.16
CG 4.2
Odisha 7.39
Maharashtra 10.732
Telangana 4.7
Goa 0
Andhra 6.72
Karnataka 8.736
A&N Islands 0.08
Lakshadweep 0.014
Tamil Nadu 8.478
Puducherry 0
Kerala 5.222
Additional CDST laboratory to be established to meet recommended population coverage (N=186)
C
Recommended number of TB Unit
Existing number TB Unit
Recommended number of CDST lab
Existing number of CDST lab
Number of TB Unit
Number of CDST laboratory
0
500
1000
1500
2000
2500
3000
3500
50
100
150
200
Andhra Pradesh
Arunachal Pradesh
Assam
Bihar
Chhattisgarh
Goa
Gujarat
Haryana
Himachal Pradesh
Jharkhand
Karnataka
Kerala
Madhya Pradesh
Maharashtra
Manipur
Meghalaya
Mizoram
Nagaland
Odisha
Punjab
Rajasthan
Sikkim
Tamil Nadu
Telangana
Tripura
Uttar Pradesh
Uttarakhand
West Bengal
D
Recommended number of TB Unit
Existing number of TB Unit
Recommended number of CDST lab
Existing number of CDST lab
Number of TB Unit
Number of CDST laboratory
0
100
200
300
5
10
15
20
A&N Islands
Chandigarh
DNHDD
Delhi
Jammu & Kashmir
Ladakh
Lakshadweep
Puducherry
E
CDST laboratory: TB Unit Ratio
0.00
0.02
0.04
0.06
0.08
0.04
0.015
0.025
61.56 %
Recommended Ratio
Existing Ratio
Gap Ratio

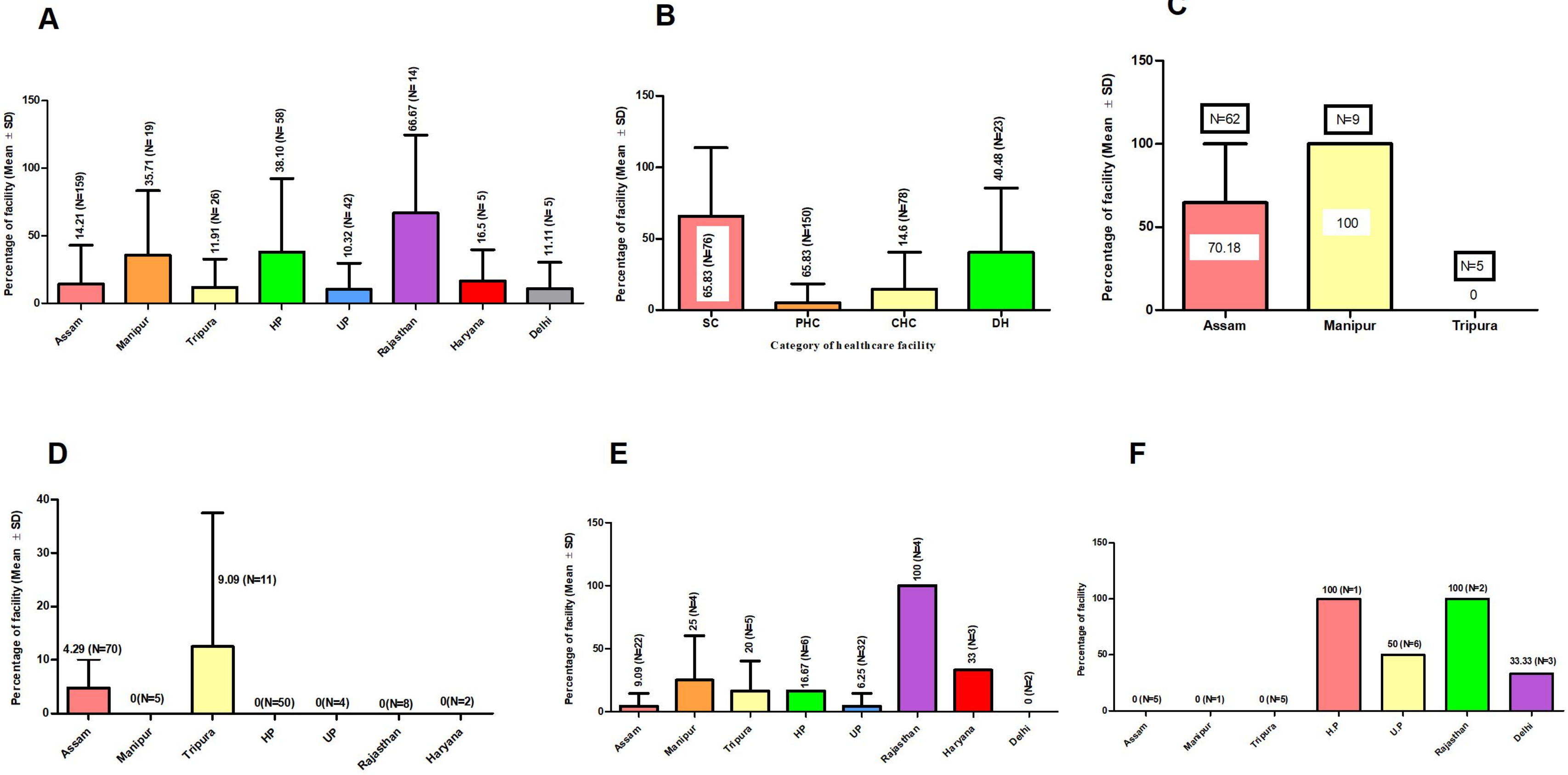

A
Percentage of facility (Mean ± SD)
150
100
50
0
14.21 (N=159)
35.71 (N= 19)
11.91 (N= 26)
38.10 (N= 58)
10.32 (N= 42)
66.67 (N= 14)
16.5 (N= 5)
11.11 (N= 5)
Assam
Manipur
Tripura
HP
UP
Rajasthan
Haryana
Delhi
B
Percentage of facility (Mean ± SD)
150
100
50
0
65.83 (N=76)
65.83 (N=150)
14.6 (N=78)
40.48 (N=23)
SC
PHC
CHC
DH
Category of healthcare facility
C
Percentage of facility (Mean ± SD)
150
100
50
0
N=62
N=9
N=5
70.18
100
0
Assam
Manipur
Tripura
D
Percentage of facility (Mean ± SD)
40
30
20
10
0
4.29 (N=70)
0(N=5)
9.09 (N=11)
0(N=50)
0(N=4)
0(N=8)
0(N=2)
Assam
Manipur
Tripura
HP
UP
Rajasthan
Haryana
E
Percentage of facility (Mean ± SD)
150
100
50
0
9.09 (N=22)
25 (N=4)
20 (N=5)
16.67 (N=6)
6.25 (N=32)
100 (N=4)
33 (N=3)
0 (N=2)
Assam
Manipur
Tripura
HP
UP
Rajasthan
Haryana
Delhi
F
Percentage of facility
150
100
50
0
0 (N=5)
0 (N=1)
0 (N=5)
100 (N=1)
50 (N=6)
100 (N=2)
33.33 (N=3)
Assam
Manipur
Tripura
H.P
U.P
Rajasthan
Delhi

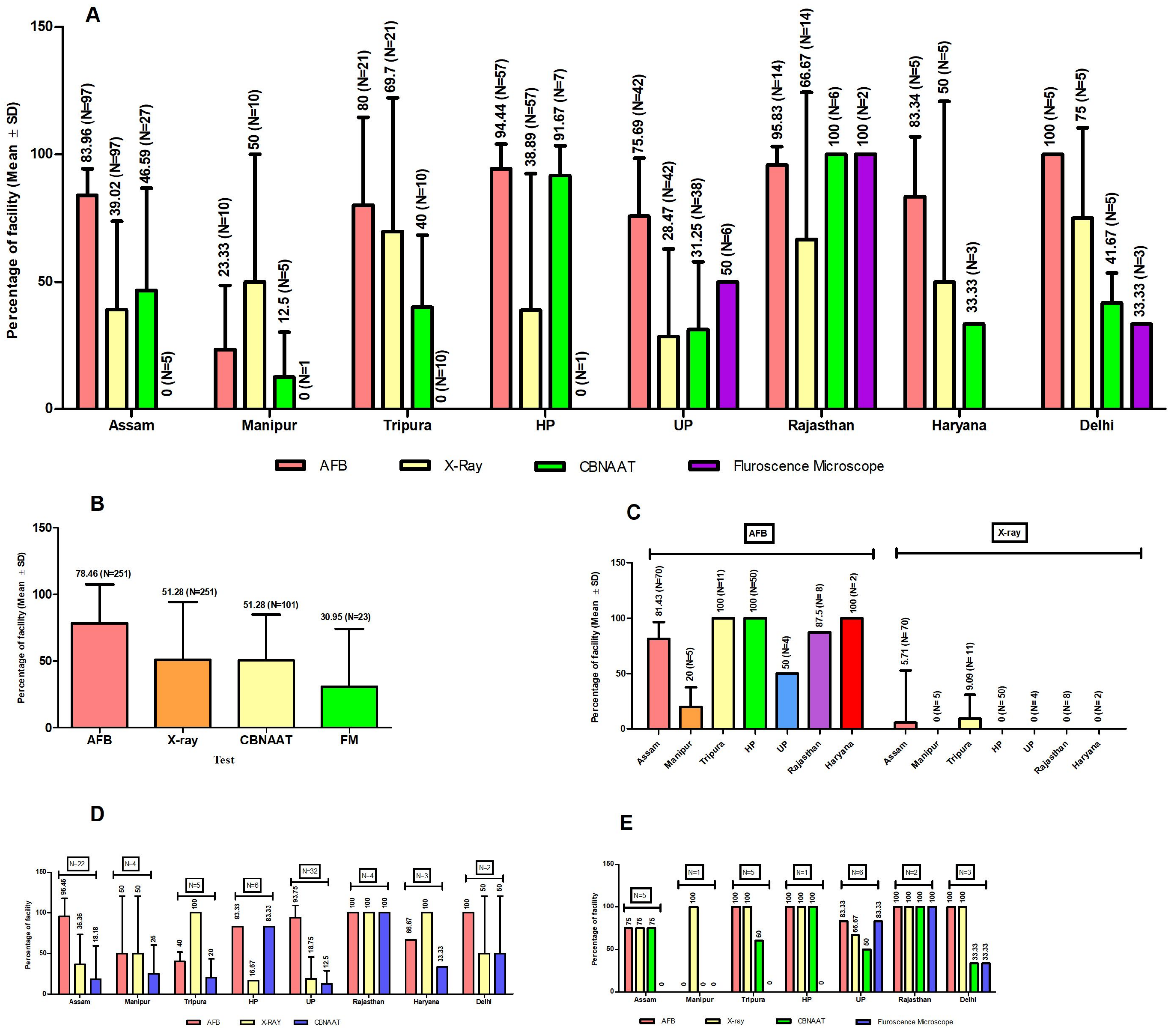

A
Percentage of facility (Mean ± SD)
150
100
50
0
83.96 (N=97)
39.02 (N=97)
46.59 (N=27)
0 (N=5)
23.33 (N=10)
50 (N=10)
12.5 (N=5)
0 (N=1
80 (N=21)
69.7 (N=21)
40 (N=10)
0 (N=10)
94.44 (N=57)
38.89 (N=57)
91.67 (N=7)
0 (N=1)
75.69 (N=42)
28.47 (N=42)
31.25 (N=38)
50 (N=6)
95.83 (N=14)
66.67 (N=14)
100 (N=6)
100 (N=2)
83.34 (N=5)
50 (N=5)
33.33 (N=3)
100 (N=5)
75 (N=5)
41.67 (N=5)
33.33 (N=3)
Assam
Manipur
Tripura
HP
UP
Rajasthan
Haryana
Delhi
AFB
X-Ray
CBNAAT
Fluroscence Microscope
B
Percentage of facility (Mean ± SD)
78.46 (N=251)
51.28 (N=251)
51.28 (N=101)
30.95 (N=23)
AFB
X-ray
CBNAAT
FM
Test
C
AFB
X-ray
Percentage of facility (Mean ± SD)
81.43 (N=70)
20 (N=5)
100 (N=11)
100 (N=50)
50 (N=4)
87.5 (N=8)
100 (N=2)
5.71 (N=70)
0 (N=5)
9.09 (N=11)
0 (N=50)
0 (N=4)
0 (N=8)
0 (N=2)
Assam
Manipur
Tripura
HP
UP
Rajasthan
Haryana
D
Percentage of facility
N=22
N=4
N=5
N=6
N=32
N=4
N=3
N=2
95.46
36.36
18.18
50
50
25
40
100
20
83.33
16.67
83.33
93.75
18.75
12.5
100
100
100
66.67
100
33.33
100
50
50
Assam
Manipur
Tripura
HP
UP
Rajasthan
Haryana
Delhi
AFB
X-RAY
CBNAAT
E
Percentage of facility
N=5
N=1
N=5
N=1
N=6
N=2
N=3
75
75
75
0
0
100
0
0
100
100
60
0
100
100
100
0
83.33
66.67
50
83.33
100
100
100
100
100
100
33.33
33.33
Assam
Manipur
Tripura
HP
UP
Rajasthan
Delhi
AFB
X-ray
CBNAAT
Fluroscence Microscope

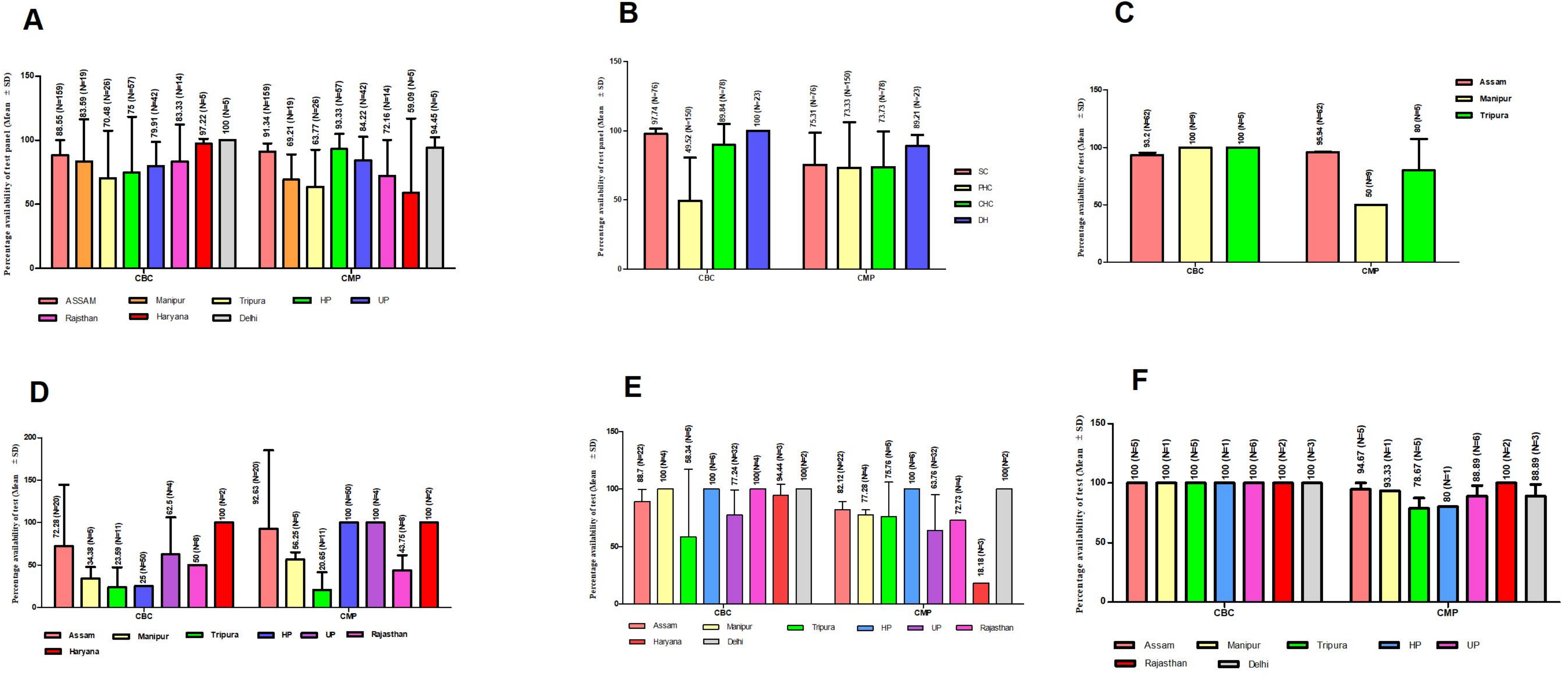

A
Percentage availability of test panel (Mean ± SD)
150
100
50
0
88.55 (N=159)
83.59 (N=19)
70.48 (N=26)
75 (N=57)
79.91 (N=42)
83.33 (N=14)
97.22 (N=5)
100 (N=5)
91.34 (N=159)
69.21 (N=19)
63.77 (N=26)
93.33 (N=57)
84.22 (N=42)
72.16 (N=14)
59.09 (N=5)
94.45 (N=5)
CBC
CMP
ASSAM
Manipur
Tripura
HP
UP
Rajsthan
Haryana
Delhi
B
Percentage availability of test panel (Mean ± SD)
150
100
50
0
97.74 (N=76)
49.52 (N=150)
89.84 (N=78)
100 (N=23)
75.31 (N=76)
73.33 (N=150)
73.73 (N=78)
89.21 (N=23)
CBC
CMP
SC
PHC
CHC
DH
C
Percentage availability of test panel (Mean ± SD)
150
100
50
0
93.2 (N=62)
100 (N=9)
100 (N=5)
95.94 (N=62)
50 (N=9)
80 (N=5)
CBC
CMP
Assam
Manipur
Tripura
D
Percentage availability of test (Mean ± SD)
200
150
100
50
0
72.28 (N=20)
34.38 (N=5)
23.59 (N=11)
25 (N=50)
62.5 (N=4)
50 (N=8)
100 (N=2)
92.63 (N=20)
56.25 (N=5)
20.65 (N=11)
100 (N=50)
100 (N=4)
43.75 (N=8)
100 (N=2)
CBC
CMP
Assam
Manipur
Tripura
HP
UP
Rajasthan
Haryana
E
Percentage availability of test (Mean ± SD)
150
100
50
0
88.7 (N=22)
100 (N=4)
58.34 (N=5)
100 (N=6)
77.24 (N=32)
100 (N=4)
94.44 (N=3)
100 (N=2)
82.12 (N=22)
77.28 (N=4)
75.76 (N=5)
100 (N=6)
63.76 (N=32)
72.73 (N=4)
18.18 (N=3)
100 (N=2)
CBC
CMP
Assam
Manipur
Tripura
HP
UP
Rajasthan
Haryana
Delhi
F
Percentage availability of test (Mean ± SD)
150
100
50
0
100 (N=5)
100 (N=1)
100 (N=5)
100 (N=1)
100 (N=6)
100 (N=2)
100 (N=3)
94.67 (N=5)
93.33 (N=1)
78.67 (N=5)
80 (N=1)
88.89 (N=6)
100 (N=2)
88.89 (N=3)
CBC
CMP
Assam
Manipur
Tripura
HP
UP
Rajasthan
Delhi

Title: Evaluating the nationwide availability of essential diagnostics for tuberculosis towards achieving a TB-free India

Brief title: Nationwide availabilty gaps of Tuberculosis Essential Diagnostics in India

Biswajit Chakraborty PhD[1*], Amaj Ahmed Laskar PhD[1*], Vipin Kumar[2], Rinki Kumar PhD[1,3], Kajal Arora MSc[4], Zeeshan Mustafa PhD[1], Anjam Hussain Barbhuiya PhD[5], Afnan Anowar Hussain MSc[5], Ashrafun Parvin MSc[5], Leichombam Mohindro Singh PhD[6], Kanu Shil PhD[7], Sumit Roy MSc[8] , Mohammad Azam Khan PhD[9], Anamika Baruah MA[1] , Mustafa A. Barbhuiya PhD[1,10,11]

[1]Foundation for Advancement of Essential Diagnostics, Bio-NEST Bio-Incubation Centre, Indian Institute of Technology, Guwahati, 781039, Assam, India.

[2]Department of Microbiology, Dr. YSP Government Medical College, Nahan, HP, 173001, India

[3]Center for Advanced Laboratory Medicine, Department of Pathology and Cell Biology, Columbia University Medical Center, New York, NY, United States.

[4]Department of Paramedical & Allied Health Sciences, School of Medical & Allied Science, Galgotias University, Uttar Pradesh, Plot No-2, Sector 17-A, Yamuna Expressway, Greater Noida, Gautam Budh Nagar, Uttar Pradesh, 203201, India.

[5]Department of Zoology, Goalpara College, Goalpara, 783101, Assam, India

[6]Microbial Resources Division, Institute Of Bio-resources And Sustainable Development (IBSD), Imphal West, Manipur, 795001, India

[7]Faculty of Paramedical Sciences, Assam Down Town University, Guwahati 781026, Assam, India.

[8]District Epidemiologist, Integrated Disease Surveillance Programme (IDSP), Karimganj, National Health Mission (NHM), Assam, India.

[9]Department of Statistics and Operations Research, Aligarh Muslim University, Aligarh, Uttar Pradesh 202001, India.

Department of [10]Pathology, [11]Healthcare Delivery and Population Sciences, UMass Chan Medical School- Baystate Regional Campus, Springfield, MA, 01199 USA

*Contributed Equally

Corresponding author:

Mustafa A. Barbhuiya, PhD
Founder and Director, Foundation for Advancement of Essential Diagnostics
Department of Pathology, Healthcare Delivery and Population Sciences
UMass Chan Medical School- Baystate Regional Campus, Springfield, MA 01199, USA
Telephone: 413-794-4512; Fax: 413-794-3195
Email: mustafa.barbhuiya@baystatehealth.org; mabarbhuiya@faed.in

Evaluating the nationwide availability of essential diagnostics for tuberculosis towards achieving a TB-free India

Abstract

India continues to face a high tuberculosis (TB) burden, as reaffirmed by the latest WHO Global TB Report. This trend highlights the need for a critical appraisal of the country's TB management framework. In this study, we assessed the distribution of TB treatment Units and Culture and Drug Susceptibility Testing (CDST) laboratories across states and Union Territories in India. The analysis revealed a pronounced national deficit of 61.56% in CDST laboratory capacity relative to projected diagnostic needs, with the gap most evident in rural and geographically remote regions. Lack of proportional availability of drug susceptibility testing or alternative molecular methods in conjunction with TB Treatment Units, leading to non-selective antibiotic use, could be the reason India has the highest multidrug-resistant TB. Additionally, our nationwide assessment of 327 healthcare facilities across seven states and the National Capital Region- Delhi revealed marked deficiencies in the implementation of Essential Diagnostics (ED) for TB within primary-level public health care facilities. Notably, access to molecular diagnostics, like Nucleic Acid Amplification Tests (NAAT), remains insufficient for early case detection, contributing to a high proportion of missed TB cases. The key TB treatment management and monitoring investigations, such as comprehensive metabolic panels and complete blood count are inconsistently available at TB Units linked to Primary and Community Health Centres. Addressing these systemic deficiencies through targeted policy action is critical to integrating essential diagnostics into routine care pathways and ensuring an accessible, high-quality TB elimination program.

1. Introduction

Tuberculosis (TB) continues to pose a significant public health challenge both worldwide and in India (1,2). Despite various national initiatives, India remains the top contributor to the global TB burden, accounting for 25% of global cases according to the World Health Organization (WHO) Global TB Report 2024 (2). Additionally, to date, there are substantial gaps between the estimated and reported numbers of TB worldwide and regionally. The WHO Global TB Report 2025 indicated that approximately 2.4 million TB cases were reported worldwide in 2024. India is a leading contributor to the TB incidence in the world. It is also the second-highest contributor, accounting for 8.8% of missing cases, after Indonesia (10%) (2). At the same time, a scary estimate of the National Prevalence Survey of India (2019-2021) reported 31 per cent tuberculosis infection burden among individuals above 15 years of age (3). However, according to the Indian government's official report, the number of missing TB cases fell from 15 lakh in 2015 to 1 lakh in 2024 (4). However, the 2025 global report challenges this claim, and many more cases likely remain undiagnosed (2). Another notable contributor to the high TB incidence in India is the rising drug-resistant strain. As per the global TB report, India accounts 32% of all multi-drug resistance (MDR)/Rifamicin resistance (RR) TB cases reported worldwide in 2024 (2). Probable challenges include a high rate of undiagnosed and untreated resistant cases, leading to poor treatment success rates and a high death rate in some cohorts (5).

The Government of India's efforts to improve TB management, including diagnosis, introduced Ni-Kshay under the National Strategic Plan (NSP), a real-time case-tracking system (6). These initiatives have improved detection and tracking. Relatedly, India's National Tuberculosis Elimination Programme (NTEP) places TB Units at the forefront of primary TB management, supported by Culture and Drug Susceptibility Testing (CDST) laboratories to detect TB infection, drug resistance, and relapse (7,8). Strengthening culture-based labs is essential for early identification of drug-resistant TB (DR-TB), which remains a bottleneck. Simultaneously, in general, to improve diagnostic accessibility, the WHO launched the Essential Diagnostics List (EDL) globally in 2018. WHO EDL aims to standardize diagnostics, reduce costs, and improve quality across all levels (9). Subsequently, India

developed its National Essential Diagnostics List (NEDL) in 2019, which includes TB diagnostics among essential diagnostics due to their significant public health impact (10). India's healthcare system is tiered—primary (Sub-Centres, PHCs), secondary (CHCs, Sub-District hospitals), and tertiary (District Hospitals/Medical Colleges) (11). Under NEDL-2019, acid-fast bacilli (AFB) smear microscopy, mobile X-ray or X-ray, Nucleic Acid Amplification Tests (NAAT) or Cartridge-Based Nucleic Acid Amplification Test (CB-NAAT), and gram staining of pus cells by Fluorescence Microscopy (FM) are included as Essential Diagnostics (ED) at public hospitals as per NEDL-2019 (10). Simultaneously, the WHO recommends NAAT or CB-NAAT for early and accurate diagnosis and has endorsed them as rapid molecular diagnostics (WRDs), which are effective for early TB detection using portable, battery-operated PCR platforms (12,13). These tools are currently listed as essential diagnostics in India only for secondary care facilities. However, only 25% of TB cases in India are diagnosed with high-sensitivity tools, probably due to limited implementation at the primary level (14). India also faces a high TB relapse rate (10%) and a TB mortality rate of 22 per lakh as of 2022 (15). This is primarily due to limited access to essential diagnostic tests for monitoring patients for treatment-associated morbidity. Drug-resistant TB requires additional monitoring during treatment (16). Despite the Indian government's efforts to introduce essential tools for TB management, the current high burden warrants re-evaluation of their implementation status at both national and local levels. Given the current context, reassessing the Indian TB management system is of paramount importance for eliminating TB in this region. Studies based on WHO EDL across ten countries revealed significant gaps in diagnostic availability at the primary care level (17). Similar findings were reported in three Indian states earlier (18). However, no comprehensive study has evaluated the availability of TB-related essential diagnostics in India, as defined by the NEDL. Thus, this study assessed the current implementation status of TB management systems in India. This includes evaluating access to TB Units, CDST laboratories, TB-specific essential diagnostics (EDs), and ED tests related to TB treatment monitoring, such as Complete Blood Count (CBC) and Comprehensive Metabolic Panel (CMP).

2. Methods

a. Design of the study

To examine potential gaps in the health system, we conducted a comprehensive assessment of the TB diagnostic and management infrastructure nationwide. In the first phase, we extracted state-level data on the number of TB Units (TUs) and on Culture and Drug Susceptibility Testing (CDST) laboratories from official, publicly available government databases. In the subsequent phase, to facilitate a root-level assessment of the basic infrastructure supporting TB management, we designed a field-level survey to evaluate the availability of essential diagnostics in accordance with NEDL-2019 across different tiers of government health facilities in select states and districts. The selection of survey sites was based on random sampling and guided by operational feasibility and resource availability. The study methodology was submitted to the institutional ethics committee and approved with reference No. NEDL/2024-2025/IEC/FAED-01.

b. Collection of data regarding the distribution of TB Units across India

Information on the number of TB Units was obtained from the Government of India's Ni-Kshay portal. District-level data on functional TB Units were collected and aggregated to calculate the total number of TB Units for each state and union territory (UT) (6). A customized map was created using an online tool (www.iipmaps.com) to visualize the current distribution of TB Units across India (19). The number of recommended TB Units for each state and Union Territory was calculated in accordance with the NTEP guideline of one TB Unit per 2 lakh population, using the most recent population estimates available on the Statistics Times website as of February 05, 2025(20,21). Finally, the gap in TB Units was calculated by comparing the recommended and existing numbers, and the results were displayed for each state and UT on a customized map of India. The details of the number of existing and recommended TB units for each state and UT are represented in Supplementary Table 1.

c. Assessment of the distribution of CDST-based TB diagnostics across India

The WHO recommends that at least one CDST laboratory be available for every five million people to ensure effective TB management (22). In this study, information on the availability of CDST laboratories across India was obtained from the India TB Report 2024 (1). The current population (in millions) for each state and union territory was obtained from the Statistics Times (www.statisticstimes.com) website (21). Gaps in CDST laboratory infrastructure coverage were then calculated using the following equations:

Equation 1:

$$Recommended\ number\ of\ CDST\ labs = \frac{Population\ (in\ millions)}{5}$$

Equation 2:

Availability Gap in CDST = Recommended CDST – Existing CDST

Equation 3:

$$CDST\ Labs{:}TB\ Unit\ Ratio\ (Recommended/Existing) = \frac{Recommended\ or\ Existing\ number\ of\ CDST\ Labs}{Recommended\ or\ Existing\ number\ of\ TB\ Units}$$

The detail state and UT-wise numbers are presented in Supplementary Table 1, showing India's current population, the existing number of CDST laboratories, the WHO-recommended number of CDST laboratories and the gaps in the number of CDST laboratories.

d. Survey of TB ED facilities in different categories of public healthcare settings

In this study, the availability of essential diagnostics (ED) for TB screening and diagnosis (Table 1) along with TB treatment monitoring diagnostic tests **(**Table 2**),** was assessed through surveys across five districts in Assam and Uttar Pradesh (UP), four in Tripura, three in Delhi, three in Manipur, and one district each in Himachal Pradesh (HP), Haryana and Rajasthan. The surveyed sites are shown in deep black color in Supplementary Figure 1. The survey focused on the availability of essential diagnostic tests for TB under different categories of healthcare facilities as recommended in the NEDL by the ICMR. The survey was conducted through in-person visits by surveyors to public health care facilities between November 2023 and April 2025. Facility staff were interviewed, and structured questionnaires (Supplementary Table 2, 3) were used. We recorded the presence or absence of diagnostic tests in different categories of healthcare facilities. This survey assessed the availability of essential TB diagnostics across seven Indian states—Assam, Manipur, Tripura, Himachal Pradesh (HP), Uttar Pradesh (UP), Rajasthan, Haryana—and one Union Territory, Delhi. The districts covered included Cachar, Sribhumi, Hailakandi, Goalpara and Kamrup Metro (Assam); South Tripura, North Tripura, West Tripura and Unakoti (Tripura); East Imphal, West Imphal and Bishnupur (Manipur); Sirmaur (HP); Gautam Budh Nagar, Aligarh, Budaun, Bulandshahr and Kasganj (UP); Jaipur (Rajasthan); Faridabad (Haryana); and Central Delhi, South West Delhi and New Delhi (Delhi) (Supplementary Figure 1). In total, we surveyed 327 public health facilities, comprising Sub-Centres (SCs, N=76), Primary Health Centres (PHCs, N=150), Community Health Centres (CHCs, N=78), and District Hospitals (DHs, N=23).

e. Statistical analysis

The study was conceived to provide the first representative, ground-level assessment of the implementation status of TB-management tools across India, using state- and UT-wise data generated

within the constraints of our operational resources. Accordingly, surveys were conducted only in locations where logistical support was available, rather than with the aim of achieving uniform state-level comparability. Consequently, the extent of coverage varied: in some states, only a single district could be surveyed, whereas in others up to five districts were included. Likewise, while certain state assessments were conducted across all four categories of health-care facilities, in others, we were able to survey only two categories. The number of facilities assessed within each category also differed considerably. These inherent asymmetries limited the scope for robust statistical comparisons across multiple datasets. However, inferential statistics plays a vital role in drawing meaningful conclusions from sample data and making generalizations about the target population. In this study, both parametric and non-parametric statistical tests are employed to ensure robustness and reliability of the results. For parametric analysis, the assumption of normality is examined; where satisfied, an independent-samples t-test is applied to compare group means. When the normality assumption is violated, or the data are ordinal, appropriate nonparametric alternatives, such as the Mann–Whitney U test and the Kruskal–Wallis test, are used to assess group differences. Furthermore, the Chi-square test is employed to assess associations and test the significance of differences between observed and expected frequencies in the data. The combined use of these statistical techniques enhances the validity of the findings and ensures that the study objectives are addressed rigorously and comprehensively.

3. Results

a. Critical Availability Gaps in TB Units and CDST Laboratories across Indian States and UTs

Ensuring sufficient TB Units and CDST laboratories is essential for effective TB control in India. Based on national benchmarks—one TB Unit per 200,000 population (NTEP) and one CDST laboratory per 5 million population (WHO)—we evaluated the existing infrastructure across all states and Union Territories (UTs). India currently has 6,308 TB Units, short of the required 7,084, resulting in a deficit of 776. Eleven states and four UTs fall below the recommended numbers, with the largest gaps in Uttar Pradesh (179), Maharashtra (167), Rajasthan (161), Karnataka (150), Kerala (125), and Bihar (113). In total, deficit regions require 1,253 additional TB Units to meet the benchmark (Figure 1A). In India, there are 97 CDST laboratories (public and private), but 283 are needed, per benchmark recommendations, resulting in a shortfall of 186. Nineteen states—including Bihar (22), Uttar Pradesh (37), West Bengal (15), Gujarat (12), Madhya Pradesh (12), Rajasthan (11), Maharashtra (11), and Odisha (7)—and one UT (Jammu & Kashmir, 2) fall below the requirement (Figure 1B).

b. Availability Gaps in CDST Laboratory to TB Unit Ratio across India

Effective TB management depends on the combined functioning of TB Units and CDST laboratories. To assess their relationship, we compared the existing numbers for each state and UT's. States showed wide statistically significant variation in both TB Units and CDST laboratories (Figure 1C). However, statistically significant variability was observed among UTs for TB Units ($\chi^2 = 73.43$, df = 6,-p value=0.0001) but non-significant difference was observed among UT's for CDST laboratories ($\chi^2 = 4.95$ with df = 7, p-value≈0.66) (Figure 1D). State- and UT-wise details on the number of TB units and CDST laboratories are provided in Supplementary Table 1. According to NTEP and WHO guidelines, one CDST laboratory is required for every 25 TB Units (i.e., a 0.04:1 ratio). Using Equation 3, we calculated the current national ratio by dividing the number of existing CDST laboratories (N=96) by the number of existing TB Units (N=6,308), yielding 0.01538. The deviation from the recommended ratio (0.04) is 0.02462, indicating a 61.56% national shortfall in CDST laboratory capacity (Figure 1E).

c. Availability of TB Essential Diagnostics as per ICMR NEDL Recommendations

According to the ICMR NEDL 2019, ED's for TB differ across these healthcare levels. The detailed availability of these diagnostics in the surveyed facilities is presented in the sections below.

i) Availability of ED for TB diagnosis across seven states and one UT

Our survey found that the availability of complete ED coverage for TB across all healthcare categories ranged from 10.32% to 66.67% across seven states and Delhi. Rajasthan had the highest availability, with 66.67% of its 14 surveyed facilities meeting all ED requirements, whereas Uttar Pradesh had the lowest, at 10.32% of its 42 facilities. Assam, Tripura, Haryana, and Delhi also reported low coverage, with 14.21% (N=159), 11.91% (N=26), 16.5% (N=5), and 11.11% (N=5) of facilities, respectively, having the full ED set. Here we performed the Kruskal-Wallis test, which indicated that there is no significant difference (p value=0.761) between states, suggesting similar implementation of facility across regions (Figure 2A). At the district level, East Imphal (N=9), Aligarh (N=12), Budaun (N=10), Jaipur (N=14), and New Delhi (N=1) had ≥50% of surveyed facilities equipped with complete EDs. Most other districts showed very low availability, and some reported none at all—including Hailakandi (N=5) and Kamrup Metro (N=3) in Assam; Unakoti (N=5) in Tripura; Bulandshahr (N=4) and Kasganj (N=4) in UP; and single surveyed facilities in four districts of Delhi (Supplementary Figure 2A). Statistical comparisons were not performed at the district level for reasons outlined in the Methods section. Overall, across the 327 surveyed facilities, SCs had the highest availability (65.83%), whereas PHCs had the lowest (4.94%). CHCs demonstrated 14.6% availability, and DHs showed 40.48% compliance. Differences across facility categories were statistically significant (p value=.029) (Figure 2B).

ii) Availability of ED for TB diagnosis across different regions and categories of health care

We surveyed ED availability for TB across 76 SCs in Assam (N=62), Manipur (N=9), and Tripura (N=5). Since sputum collection is the only SC-level ED under ICMR NEDL-2019, our analysis focused on this service. All SCs in Manipur had sputum collection, compared with 70% in Assam and 0% in Tripura (Figure 2C). At the district level, Sribhumi and both Imphal districts showed 100% availability, whereas Cachar had 29.17%, and South Tripura had none (Supplementary Figure 2B). At the PHC level (N=150), ED availability was defined by the presence of AFB microscopy and mobile or standard X-ray. ED coverage was consistently low, ranging from 0% to 10%. No PHCs in Manipur, Himachal Pradesh, Uttar Pradesh, Rajasthan, or Haryana had the required diagnostics. Limited availability was observed in Assam (4.29%) and Tripura (9.9%) (Figure 2D). District-level variation is shown in Supplementary Figure 2C. At the CHC level (N=78), EDs include AFB microscopy, X-ray, and CBNAAT. Overall availability remained low (0–35%). Coverage was 9.09% in Assam, 25% in Manipur, 20% in Tripura, 16.67% in Himachal Pradesh, 6.25% in Uttar Pradesh, 33% in Haryana, and 0% in Delhi. Rajasthan was the only state where all four surveyed CHCs had complete EDs (Figure 2E). District-level availability ranged from 100% in Jaipur to 0% in 12 districts (Supplementary Figure 2D). At the District Hospital (DH) level, the four required EDs under NEDL-2019 include AFB microscopy, fluorescence microscopy for pus cells, X-ray, and CBNAAT. Complete ED availability was absent in all surveyed DHs in Assam (N=5), Manipur (N=1), and Tripura (N=5). In contrast, one DH in Himachal Pradesh, two in Rajasthan, and one of three in Delhi met all requirements (Figure 2F). The statistical analysis for the data set of Figure 2C to Figure 2F and district-level percentage availability of ED (Supplementary Figure 2B to Supplementary Figure 2D) could not be performed for the reasons detailed in the Methods section.

d. Availability of each ED test for TB diagnosis across different region and category of health care

The AFB smear test, an essential diagnostic (ED) for PHCs, CHCs, and DHs, was the most widely available test compared to X-ray, CBNAAT, and FM. Its lowest availability was in Manipur (23.33%, N=10), while in the other six states and Delhi, 75–100% of facilities had it (Figure 3A). X-ray availability was also poor across all states, with none reaching 50% operational coverage; Uttar Pradesh showed the lowest availability (28.47%, N=42). CBNAAT, recommended for CHCs and DHs, was also limited, except in Himachal Pradesh (91.67%, N=7) and Rajasthan (100%, N=6). FM for pus-cell detection, recommended only for DHs, showed highly variable availability, with no DHs in Assam, Tripura, or Manipur having the test; Rajasthan (100%, N=2) and Uttar Pradesh (50%, N=6) had the highest availability. Delhi showed moderate presence (33.33%, N=3). Resource constraints limited the number of DHs surveyed (N=23). The Kruskal–Wallis test indicated a statistically significant difference (p value=0.035) among the tests across different regions.

Overall, after assessing the percentage availability of facilities offering each diagnostic test—aggregated across categories of health-care facilities and regions—we found that AFB microscopy was available in 78.46% of recommended facilities, X-ray services in 51.28% of 251 PHCs/CHCs/DHs, CBNAAT in 51.28% of 101 CHCs/DHs, and FM in only 30.95% of 23 DHs. The differences in availability among these tests were statistically significant (p-value=0.029), as indicated by the Kruskal–Wallis test (Figure 3B).

In the health care category-wise comparison of tests, SCs were excluded because NEDL-2019 recommends only sputum collection as the ED for TB at this level (Table 1), which does not fall under any test. In PHCs, AFB availability ranged from 50–100% across states except Manipur (20%, N=5), whereas X-ray availability was uniformly low (0–10%) (Figure 3C). Formal statistical testing was not feasible owing to the limited number of district-level replicates for PHC-wise comparison, as outlined in the Methods section.

District-wise PHC data appear in Supplementary Figure 3A and the difference was found be significant (p value=0.0001) by Mann Whitney U test. Across CHCs, most states and Delhi had 80–100% AFB availability, except Manipur (50%, N=4). X-ray availability varied widely, with particularly low rates in Uttar Pradesh (18.75%, N=32) and Himachal Pradesh (16.67%, N=6). CBNAAT availability was highest in Himachal Pradesh (83.33%, N=6), Delhi (50%, N=2), and Rajasthan (100%, N=4), while the remaining states had only 10–40% coverage (Figure 3D). No statistical analysis could be performed due to the limited number of district-level replicates available for CHC-wise comparison, as described in the Methods section.

District-wise CHC details are provided in Supplementary Figure 3B, and the Kruskal-Wallis test showed significant differences (p value=0.001) among them. At the DH level, the availability of AFB, X-ray, and CBNAAT was generally highest across states and Delhi, except in Manipur, where only AFB was available. FM availability remained low in Assam, Tripura, Manipur, and Delhi, but was highest in Rajasthan (100%, N=2) and Uttar Pradesh (83.33%, N=6) (Figure 3E). Statistical analysis was not performed due to limitations described in the methods.

e. Regional and Facility-Level Availability of Essential Diagnostics Related to CBC and CMP Panels

The CBC and CMP test panel are essential for TB treatment and management. We assessed their availability across 327 healthcare facilities in seven states and one Union Territory. The CBC panel included six tests (RBC, WBC/TLC, platelets, hemoglobin, hematocrit, and Mean Corpuscular

Volume test), and the CMP panel included 15 tests, as recommended under ICMR–NEDL 2019 (Table 2). Based on these guidelines, we calculated the percentage availability of required tests in each facility. Across states and Delhi, CBC availability ranged from 70–100%, with Tripura lowest (70.48%, N=26). CMP availability showed a similar pattern (59–95%), with Haryana having the lowest value (59.09%, N=5). For statistical testing, we use an independent sample t-test, which showed no significant differences (p-value=0.322) between the CBC and CMP test panels across states (Figure 4A).

By facility type, CBC availability was high in SCs, CHCs, and DHs (89–100%) but much lower in PHCs (49.52%, N=150). CMP availability was 73–76% in SCs, PHCs, and CHCs, and highest in DHs (89.21%, N=23), with statistically non-significant differences (p value=0.248) between CBC and CMP test panels across various healthcare centres were indicated by the Mann-Whitney U test (Figure 4B).

State-level CBC availability in SC's from Assam, Manipur, and Tripura was 93–100%, while CMP availability was lowest in Manipur (50%, N=9), also from Mann-Whitney U test we find that there is no significant difference (p value=.121) CBC and CMP test panels in SC's of these three states (Figure 4C). At the district level, CBC availability was lowest in West and East Imphal (both 50%) (Supplementary Figure 4A). Mann-Whitney U test showed no significant differences (p value=.071) CBC and CMP test panels among SCs across states or districts.

Among PHCs, CBC availability was low in HP (25%), Tripura (23.59%), and Manipur (34.38%), whereas other states performed better. CMP availability at PHCs was generally higher than CBC, except in Tripura (20.65%) (Figure 4D). From independent sample t-test, we observe that there is no significant difference (p value=.226) in the availability of CBC and CMP test panels in PHC's of states. Also Mann-Whitney U showed statistically significant difference (p-value=0.010) between CBC and CMP test panels among PHC's of district-level (Supplementary Figure 4B).

In CHCs, CBC availability was lowest in Tripura (58.34%, N=5) and 75–100% elsewhere. CMP availability was 70–100% across most states but very low in Haryana (18.18%, N=3) (Figure 4E). District-level CMP availability was particularly low in West Tripura, Budaun, and Faridabad (Supplementary Figure 4C). Independent sample t-test showed no significant difference (p-value=0.149) between CBC and CMP test panels across states, while the Mann-Whitney U test showed a significant difference (p-value=0.010) between CBC and CMP test panels across districts.

At the DH level, CBC availability was 100% in all states and Delhi. CMP availability was also high, except for slightly lower values in Tripura (78.67%, N=5) and Uttar Pradesh (88.89%, N=6) (Figure 4F). There is a significant variation (p-value=.004) in the availability of CBC and CMP test panels across states, as observed at the DH level, as indicated by the Mann-Whitney U test.

4. Discussion

Tuberculosis (TB) continues to pose a profound public health challenge in India, despite sustained governmental efforts aimed at curbing its burden. Although several strategic interventions have been initiated under the NTEP, the current Global TB Report indicates that these measures remain insufficient to meaningfully alter the disease trajectory. India accounts for one-fourth of the global TB burden, underscoring the urgency of reassessing national strategies and strengthening the implementation of existing diagnostic and management tools (2). The Government of India's aspiration to eliminate TB by 2025—an objective that is ambitious and commendable—appears increasingly unattainable when contrasted against prevailing epidemiological realities (23).

Multiple, interrelated factors contribute to India's high TB burden. These include the country's vast and diverse population, high rates of missed diagnoses, inconsistent implementation of programme guidelines, and inadequate detection and monitoring of drug-resistant TB strains (24). Early and accurate detection is the most critical determinant for infectious disease control. However, India

remains the second-largest contributor to the world's 2.4 million "missing TB cases," accounting for 8.8% of the global total (2). Although national reports indicate improvements in case detection rates, such aggregated numbers mask substantial heterogeneity across states, districts, and population groups (4). For example, TB incidence among tribal communities has been reported at 703 per 100,000 population—more than threefold higher than the national estimate of 199 per 100,000 (25). This stark contrast reflects deep-rooted socio-economic inequities, including poverty, stigma, illiteracy, and chronic infrastructural deficiencies in rural and tribal regions. Ensuring equitable implementation of TB control strategies across these marginalized populations is therefore imperative. The problem is further compounded by escalating levels of drug-resistant TB (DR-TB). As highlighted in the World TB Report 2025, India accounts for approximately 32% of global drug-resistant TB cases—the highest worldwide (2). Such alarming levels of resistance are likely driven by inappropriate antibiotic usage, inconsistent adherence to treatment protocols, and inadequate deployment of advanced molecular diagnostic tools capable of detecting resistance patterns early (26). The persistence of these challenges indicates structural gaps in health system capacity, particularly at the primary and secondary levels where initial diagnosis and treatment initiation occur (27). Within the organizational architecture of TB control in India, TB units constitute the first operational and administrative tier. TU's are responsible for diagnosing, treating, and tracking TB cases within defined geographical areas. Complementing these units are CDST laboratories, which provide specialized diagnostic services required to identify drug resistance and guide complex treatment regimens. TUs typically collect samples from presumptive TB patients and refer them to CDST laboratories for confirmation and resistance testing. Thus, TUs and CDST laboratories together form the backbone of India's diagnostic referral system and play a pivotal role in ensuring the timely, accurate, and programmatically relevant flow of data under NTEP.

Moreover, according to WHO guidelines, delayed and incomplete detection of drug resistance contributes substantially to the emergence and transmission of multidrug-resistant TB (MDR-TB) (12). While WHO-endorsed PCR-based molecular assays allow rapid detection of MTB and selected resistance-associated mutations, they are limited to predefined genetic targets and may miss rare or novel mutations. Moreover, no widely available comprehensive multidrug PCR panel exists to detect resistance to all first- and second-line anti-TB drugs, and molecular assays do not provide phenotypic susceptibility data. Therefore, WHO continues to recommend culture-based drug susceptibility testing (CDST) as the reference standard for confirming resistance and guiding individualized treatment in drug-resistant TB (28,29).

In this context, our study sought to evaluate the existing infrastructural landscape for TB diagnosis and management across the country. We extracted the current number of TUs from the Nikshay portal and the number of CDST laboratories from the India TB Report 2024 for all Indian states and UT's. Based on the NTEP recommendation of one TU per 200,000 population and population estimates from Statistics Times till 5th February, 2025, we calculated the state-wise and national gaps in TU availability. Our findings reveal substantial disparities. While a few states have more TUs than recommended, most fall significantly short of the required number. This uneven distribution suggests a concentration of TUs in urban and semi-urban locations, leaving rural, remote, and socio-economically disadvantaged regions underserved. A similar pattern was observed in the distribution of CDST laboratories, with several states lacking adequate laboratory capacity to support rapid and accurate diagnosis of drug-resistant TB. Aggregating state-level requirements indicates that India requires an additional 1,215 TUs and 176 CDST laboratories to meet population-based recommendations. At the national scale, this translates to an unmet requirement of 776 TUs and 187 CDST laboratories. Moreover, the national ratio of CDST laboratories to TUs reveals a 62.5% deficit, highlighting a significant imbalance that may impede timely referral, diagnosis, and treatment of drug-resistant TB cases (Figure 1 and Supplementary Table 1). These findings collectively reaffirm the critical need for expanding diagnostic infrastructure, particularly in rural and geographically challenging regions where TB detection is often delayed or entirely missed.

To further understand the factors underpinning widespread under-diagnosis, we examined the availability and implementation of ED's for TB across different categories of public health facilities. TUs often function within or in coordination with PHCs or CHCs. Therefore, understanding the diagnostic capacity of these facilities is central to evaluating the broader TB control apparatus. India operates a three-tier public health system: primary care (SC's and PHCs), secondary care (CHCs and DHs), and tertiary care (specialist and teaching hospitals). Each tier plays a distinct role in service delivery (10). Primary-level facilities serve as the first point of contact for rural populations, secondary facilities provide more advanced diagnostic and treatment services, and tertiary institutions manage complex and severe cases.

To evaluate TB-specific ED availability at each level, we conducted a field survey covering 327 facilities across seven states—Assam, Manipur, Tripura, HP, UP, Rajasthan, Haryana—and the Union Territory of Delhi. According to ICMR's NEDL-2019, four key tests constitute the diagnostic foundation for TB detection: AFB smear microscopy (both conventional and fluorescence-based), chest X-ray, and NAAT such as CBNAAT or chip-based microPCR (11). Among these, AFB smear microscopy is the most basic yet widely recommended method due to its affordability and minimal infrastructural requirements. Chest X-ray is another critical tool, essential for supporting the diagnosis of both pulmonary and extrapulmonary TB. Despite these recommendations, our survey revealed significantly low availability of AFB microscopy and chest X-ray facilities, particularly at PHCs and CHCs. This suggests that presumptive TB patients in rural areas are often required to travel considerable distances to DHs for basic diagnostic services—leading to delays, increased financial burden, and higher likelihood of patient dropout. The situation is even more concerning with regard to CBNAAT, a WHO-endorsed rapid molecular diagnostic tool that offers high sensitivity and specificity and is now considered essential for initial testing of all presumptive TB cases. Globally, only one-third of TB patients are diagnosed using WHO-recommended rapid diagnostics (WRDs). In India, this proportion is below 25%, reflecting systemic shortfalls in the deployment of advanced diagnostic technologies where they are most needed (14). Notably, NAAT-based tests are not currently recommended for PHCs in India, limiting their accessibility to large sections of the population. Our survey results clearly show that the availability of TB-essential diagnostics across the studied states and Delhi is poor, with PHCs and CHCs exhibiting particularly serious deficiencies. While DHs demonstrate comparatively better availability, the lack of diagnostic services at lower tiers of care results in overwhelming patient loads and further delays in diagnosis. The district-wise distribution of diagnostics reveals substantial geographical disparities, particularly in regions characterized by difficult terrain and high proportions of tribal populations. These areas face disproportionately high risks of missed diagnoses and undetected transmission.

To complement our evaluation of TB-specific diagnostics, we assessed the availability of two fundamental laboratory tests—CBC and the CMP test panel—that are essential for monitoring TB patients, especially during treatment. Anemia and thrombocytopenia are commonly reported hematological abnormalities in patients during and after treatment for tuberculosis (TB). Anemia in TB is multifactorial and is primarily related to chronic inflammation, iron sequestration, nutritional deficiencies, bone marrow suppression, and adverse effects of anti-tubercular therapy (30,31). Thrombocytopenia, although less frequent, has been observed during TB treatment and may result from immune-mediated mechanisms or drug-induced toxicity, particularly associated with rifampicin and isoniazid (32,33). Rifampicin-induced immune thrombocytopenia is a recognized but potentially severe complication that can occur during prolonged or intermittent therapy (33). Persistent cytopenias during or following TB treatment require careful clinical evaluation to distinguish drug-related adverse effects from ongoing inflammation or underlying hematological disorders.

Given that many TUs are associated with PHCs or CHCs, the availability of these essential diagnostic tests is crucial. Our findings indicate that PHCs have less access to CBC and CMP testing. Although NEDL-2019 recommended only two tests under the CMP test panel for PHCs, their availability remains inadequate. Additionally, the recent NEDL-2025 version proposes expanding the list of tests for PHCs (34); however, such an expansion is unlikely to have a meaningful impact unless all essential

diagnostic tests are first made universally available. However, our survey indicates that CBC and CMP availability is better than that of TB-specific EDs, yet remains insufficient to support comprehensive TB management. Above all, to fight against multi-drug-resistant TB and the emergence of new drug resistance variants calls for a strong CDST laboratory network that is visibly lacking as per the findings in this study. PCR- and sequencing-based tools have their place, but CDST labs remain the backbone of these methods and cannot be ignored.

Achieving TB elimination in India will require optimizing primary health care infrastructure, particularly by systematically scaling up NAAT-based diagnostics for early detection and by improving the availability of CMP testing for treatment monitoring. In our previous review article, we extensively analysed a range of diagnostic recommendations for TB diagnosis derived from international agency guidelines, and underscored the importance of revising national diagnostic policies to align with country-specific needs (35). Moreover, the launch of the "100-Day TB Elimination Mission" by the Union Ministry of Health and Family Welfare on December 6, 2024, reflects renewed national commitment toward improving case detection, reducing delays, and enhancing treatment outcomes—especially among vulnerable populations (36). By presenting extensive field-level data on diagnostic facility availability across multiple tiers of public health services, our analysis provides critical insights to support more targeted and effective implementation of India's TB elimination strategy.

5. References

1. India TB Report 2024. Central TB Division, Ministry of Health and Family Welfare, Government of India, (2024).
2. Global tuberculosis report 2025. Geneva: World Health Organization, (2025).
3. Chauhan, A. et al. The prevalence of tuberculosis infection in India: A systematic review and meta-analysis. Indian J Med Res 157(2&3),135-151 (2023).
4. TB incidence in India drops by 21% from 237 per lakh population in 2015 to 187 per lakh population in 2024 – almost double the rate of decline observed globally. Reference Number: HFW-WHO TB Report/12th Nov 2025/3. Ministry of Health and Family Welfare, Government of India, (2025).
5. Bloom, B.R. et al. Tuberculosis. In: Holmes, K.K. et al., editors. Major Infectious Diseases. 3rd edition. Washington (DC): The International Bank for Reconstruction and Development / The World Bank, Nov 3. Chapter 11 (2017).
6. Narayanappa, S., Shah, H., Ps, B., Patel, J.H.S. & Saxena, D. Exploring the Ni-kshay SETU e-Platform Ecosystem: A Mixed-Methods Study on Strengthening Capacity Building Training for TB Care and Prevention (STEP) Among Interns. Cureus 15, 17(6):e86092 (2025).
7. Evolution of TB Elimination Programme in India. Tuberculosis Unit; Central TB Division, Ministry of Health and Family Welfare, Government of India.
8. Knowledge base, Culture Drug Susceptibility Testing [CDST] of NTEP. Central TB Division, Ministry of Health and Family Welfare, Government of India.
9. Bagcchi, S. WHO launches Essential Diagnostics List. Lancet Infect Dis. 2018 18(7),724-725 (2018).
10. Chokshi, M., et al. Health systems in India. J Perinatol 36(s3), S9-S12 (2016).
11. National Essential Diagnostics List-2019, Indian Council of Medical Research, Govt. of India, New Delhi, 2019.
12. WHO consolidated guidelines on tuberculosis: module 3: diagnosis: tests for TB infection. 30th. Geneva: World Health Organization September, (2023).
13. Balisan. O.P.R., Galamay, J.R.T., Cale-Subia, L.N. & De Luna, A.M. Utility of nucleic acid amplification test in the detection of tuberculosis in biological fluids from suspected TB patients in a cardiovascular center in the Philippines. Acta Trop 249:107078 (2024).
14. Hashim, Z., Tyagi, R., Singh, G.V, Nath A, Kant S. Preventive treatment for latent tuberculosis from Indian perspective. Lung India 1, 41(1), 47-54 (2024).

15. Azhar, G.S. DOTS for TB relapse in India: A systematic review. Lung India 29(2), 147-53 (2012).
16. Clinical Overview of Drug-Resistant Tuberculosis Disease. Centers for Disease Control and Prevention (CDC), Atlanta, USA. 6th January (2025).
17. Yadav, H., Shah, D., Sayed, S., Horton, S. & Schroeder, L.F. Availability of essential diagnostics in ten low-income and middle-income countries: results from national health facility surveys. Lancet Glob Health 9(11), e1553-e1560 (2021).
18. Kohli. M., Walia, K., Mazumdar, S., Boehme, C.C., Katz, Z. & Pai, M. Availability of essential diagnostics in primary care in India. Lancet Infect Dis 18(10), 1064-1065 (2018).
19. Internet Imaging Protocol Maps. Website link: https://iipmaps.com/.
20. Tuberculosis Unit, Knowledge base. National Tuberculosis Elimination Programme (NTEP). Central TB Division (CTD), Ministry of Health and Family Welfare, Government of India.
21. Population of Indian states, Statistics times, India. Website link: https://www.statisticstimes.com/demographics/india-statistics.php.
22. Laboratories providing tuberculosis diagnostic services using culture (per 5 million population). Geneva: World Health Organization.
23. Sachdeva, K.S. TB free India by 2025: hype or hope. Expert Rev Respir Med 2021 15(7), 863-865 (2021).
24. Selvaraju, S., et al. Prevalence and factors associated with tuberculosis infection in India. J Infect Public Health 16 (12), 2058-2065 (2023).
25. Satav, A., et al. Systematic review of barriers to and enablers of tuberculosis diagnosis, notification, and intervention for designing customised intervention package to minimise 'missing millions' in tribal communities of India. J Glob Health 14, 15:04303 (2025).
26. Taneja, N. & Sharma, M. Antimicrobial resistance in the environment: The Indian scenario. Indian J Med Res 149(2), 119-128 (2019).
27. Husain, A.A., Kupz, A. & Kashyap, R.S. Controlling the drug-resistant tuberculosis epidemic in India: challenges and implications. Epidemiol Health 43:e2021022 (2021).
28. Pai, M. & Nicol, M.P. Tuberculosis diagnostics: state of the art and future directions. Microbiol Spectrum 3(2), 1–15 (2015).
29. Lawn, S.D. & Nicol, M.P. Xpert MTB/RIF assay for TB and rifampicin resistance. Future Microbiology 6(9), 1067–1082 (2011).
30. Weiss, G. & Goodnough, L.T. Anemia of chronic disease. New England Journal of Medicine 352(10), 1011–1023 (2005).
31. Morris, C.D., Bird, A.R. & Nell, H. The haematological and biochemical changes in severe pulmonary tuberculosis. Quarterly Journal of Medicine 73(272), 1151–1159 (1989).
32. George, J.N. & Aster, R,H. Drug-induced thrombocytopenia: pathogenesis, evaluation, and management. Hematology Am Soc Hematol Educ Program.153–158, (2009).
33. Sharma, A., Kumar, R. & Sharma, S.K. Rifampicin-induced immune thrombocytopenia. Indian Journal of Medical Science 57(10), 456–459 (2003).

34. National Essential Diagnostics List-2025, Indian Council of Medical Research, Govt. of India, New Delhi, (2025).
35. Chakraborty, B., Laskar, A.A., Baruah, A., Kumar, R. & Barbhuiya, M.A. Critical Perspectives on Global Health Policy, Diagnostic Access and Practice Gaps towards a Tuberculosis Free India. Ann Clin Lab Sci 54(6), 721-738 (2024).
36. India’s 100-Day TB Elimination Campaign; A Decisive Step Towards Ending Tuberculosis. Ministry of Health and Family Welfare, Government of India. 06 DEC (2024).

Table 1. List of TB Essential Diagnostic Tests Recommended for Different Health Centres as per ICMR NEDL, 2019.

Sl. No.	Category of Health Centres	Test Recommended	Specimen Type	Equipment Required
**1**	Village Level Health Centre	Sample collection for Acid- Fast Bacilli (AFB) Smear Microscopy Test	Sputum in disposable sterile containers	Sample to be sent to PHC for Microscopy
**2**	Sub-Centre (SC)	Sample collection for AFB Smear Microscopy Test	Sputum in disposable sterile containers	Sample to be sent to PHC for Microscopy
**3**	Primary Health Centre (PHC)	AFB Smear Microscopy Test	Sputum in disposable sterile containers	Microscopy (ZN stain)
		X-ray for TB Detection	Chest X-ray	Referral to nearest CHC
		Mobile X-ray		
**4**	Community Health Centre (CHC)	AFB Smear Microscopy Test	Sputum in disposable sterile containers	Microscopy (ZN stain)
		Chip based Real time micro-PCR Test	Sputum in disposable sterile containers	Micro PCR
		X-ray for TB Detection	Chest X-ray	X-ray Machine
		Sample collection for Nucleic Acid Amplification Test (NAAT)	Sputum in disposable sterile containers	Sample to be sent to DH for NAAT Test
**5**	Sub-District Hospital (SDH)	AFB Smear Microscopy Test	Sputum, body fluids	Microscopy (ZN stain)
		X-ray for TB Detection	Chest X-ray	X-ray Machine
		Chip based Real time micro-PCR Test	Sputum in disposable sterile containers	Micro PCR
		Sample collection for NAAT	Sputum in disposable sterile containers	Sample to be sent to DH for NAAT Test
**6**	District Hospital (DH)	Sputum for AFB Microscopy Test<br>Pus for Gram Staining	Sputum, body fluids	Fluorescence Microscopy
		Sputum Cytology	Sputum	Microscopy
		X-ray for TB Detection	Chest X-ray	X-ray Machine
		NAAT	Sputum	Truenat/Gene Xpert (PCR based)
		Sample collection for TB culture (liquid)	Sputum, fluid etc.	Sample to be sent to nearest TB Lab/State TB Lab
		Sample collection for TB Drug Susceptibility Test (DST)	Sputum, fluid etc.	Sample to be sent to nearest TB Lab/State TB Lab

Table 2: **Components of Complete Blood Count (CBC) and Comprehensive Metabolic Panel (CMP) Essential Diagnostic Tests Recommended for Different Health Centres as per ICMR NEDL, 2019. Useful for TB treatment monitoring**

Sl. No.	Category of Health Centres	Test Recommended
	SC	**CBC**
1		Hemoglobin
		**CMP**
1		Blood sugar
2		Albumin
	PHC	**CBC**
1		Hemoglobin
2		Red blood cells count-
3		Total Leucocyte Count or WBC count
4		Platelets count
		**CMP**
1		Blood Sugar
2		Albumin
	CHC	**CBC**
1		Hemoglobin
2		Red Blood cells count
3		Total Leucocyte Count or WBC count
4		Platelets count
5		Hematocrit count
6		Mean Corpuscular Volume (MCV)
		**CMP**
1		Albumin
2		Blood sugar
3		Total bilirubin
4		Aspartate aminotransferase (AST) or SGOT
5		Alanine aminotransferase (ALT) or SGPT
6		Alkaline phosphatase
7		Creatinine

8		Blood urea
9		Sodium
10		Potassium
11		Calcium
		**CBC**
1		Hemoglobin
2		Red Blood cells count
3		Total Leucocyte Count or WBC count
4		Platelets count
5		Hematocrit count
6		Mean Corpuscular Volume (MCV)
		**CMP**
1		Albumin
2		Blood sugar
3		Total bilirubin
4	DH	Aspartate aminotransferase (AST) or SGOT
5		Alanine aminotransferase (ALT) or SGPT
6		Alkaline phosphatase
7		Creatinine
8		Blood urea
9		Sodium
10		Potassium
11		Calcium
12		Total protein
13		Blood Urea Nitrogen (BUN)
14		Chlorides
15		Blood Gas analysis or bi-carbonate

Figure Legends

Figure 1: Gaps in the number of TB Units and CDST laboratories across Indian States and Union Territories (UTs) are shown in customized maps. **A. TB Units:** The national gap of 776 TB Units was calculated by subtracting the existing 6,308 units from the recommended 7,084 units, with state-wise and UT-wise gaps also represented (Supplementary Table 1). **B. CDST laboratories:** The maps also depict the national shortfall of 186 CDST laboratories, derived from the difference between the existing 97 laboratories and the recommended 283, along with corresponding gaps for each state and UT (Supplementary Table 1). **C. State-wise representation:** The existing number of TB Units in each state was compiled from district-level data in the NIKSHAY portal, while the recommended number was estimated using the NTEP norm of one TB Unit per 2 lakh population based on the February 05, 2025, population data. Existing CDST laboratory numbers were taken from the India TB Report 2022, and the recommended numbers were calculated using the WHO guideline of one CDST laboratory per 5 million population. A chi-square t-test showed significant gaps between recommended and existing TB Units (P=0.0001, $\chi2$=566.93, df=27) and CDST laboratories (P=0.001, $\chi2$=144.44, df=27) (Supplementary Table 1). **D. UT-wise representation:** Data collection and calculations followed the same approach as for states. A chi-square test indicated a significant difference between recommended and existing numbers of TB Units (P=0.0001, χ^2 = 73.43, df=6) or CDST laboratories (P=0.66, χ^2 = 4.95, df=6) (Supplementary Table 1). **E. Nationwide ratio:** The CDST laboratory-to-TB Unit ratio was calculated using existing facility numbers, while the recommended ratio was derived using guideline-based estimates (1 CDST per 25 TB Units; ratio 0.04). The percentage gap was computed by comparing the existing ratio with the recommended ratio.

Figure 2: Availability of TB Essential Diagnostics (TB-ED) in percentage of facilities across states and categories of healthcare facilities. A. Percentage of facilities with complete TB-ED across states: The graph presents the mean ± SD percentage of facilities with cumulative values for SCs, PHCs, CHCs, and DHs that have all NEDL-2019–listed TB diagnostic tests required for their category. ED availability was counted only when every required test was present, and "N" represents the number of facilities being surveyed. A chi-square test showed a significant difference in ED availability between states (P=0.01, $\chi2$=4.16, df=7). **B. Percentage of facilities having TB-ED across different categories of healthcare facilities.** This graph represents the ED availability in percentage of the facility in mean ± SD according to the category of health care by cumulating state-level values. ED availability was counted only when every required test was present, and "N" represents the number of facilities being surveyed. Kruskal-Wallis test showed a significant difference in the percentage of facilities having complete ED according to the category of health care (P=0.001, $\chi2$=9.01, df=3).**C. Percentage of SC having TB-ED across different states.** In Sub-Centres (SCs), sputum collection is the only Essential Diagnostic (ED) included for TB diagnosis (Table 1). In this figure, we show the mean ± SD percentage of SCs that have sputum collection facilities. District-level percentages were treated as replicates. We did not perform statistical tests because, in Tripura, surveys were conducted in only one district; therefore, no replicates were available. The "N" indicates the number of SCs surveyed. **D. Percentage of PHCs having TB-ED across different states.** Two tests—AFB smear by microscopy and X-ray—are included as ED's for TB diagnosis at PHCs (Table 1). In this figure, ED availability for a PHC is shown only when both tests are present. For states with more than one district surveyed, district-level percentages of PHCs with ED availability were treated as replicates and reported as the state mean ± SD. Statistical analysis was not performed to compare PHC values across states because, in Himachal Pradesh (Sirmaur), Uttar Pradesh (Gautam Buddha Nagar), Rajasthan (Jaipur), and Haryana (Faridabad), only one district was surveyed in each state, leaving no replicates. **E. Percentage of CHCs having TB-ED across different states and Delhi.** Three tests—AFB smear by microscopy, X-ray, and CBNAAT—are included as ED for TB diagnosis at CHCs. The figure shows ED availability only when all three tests are present in a CHC. State-wise values are

presented as mean ± SD, using district-level availability as replicates for each state or UT. No statistical analysis was performed for the same reasons described for SCs and PHCs. **F. Percentage of DH's having TB-ED across different states and Delhi.** Four tests—AFB smear by microscopy, Gram staining of pus cells by fluorescence microscopy, X-ray, and CBNAAT—are included as Essential Diagnostics (EDs) for TB diagnosis at District Hospitals (DHs). The figure shows ED availability only when all four tests are present in a DH. State-wise values are presented as the percentage of DHs with complete ED availability. Statistical analysis was not performed because each district typically has only one DH, leaving no replicates; therefore, only percentages could be reported.

Figure 3: Availability of each essential diagnostic test for TB diagnosis in percentage of facility across states and categories of healthcare facilities. A. Availability of each essential diagnostic test for TB diagnosis across the state and UT Delhi. Four tests—AFB smear by microscopy, Gram staining of pus cells by fluorescence microscopy, X-ray, and CBNAAT—are included as Essential Diagnostics (EDs) for TB diagnosis across healthcare facility categories. This figure presents the cumulative percentage of facilities across all categories that have each test available in every state and UT. Results are expressed as mean ± SD, and "N" indicates the number of facilities surveyed. The differences in test-wise availability across the selected regions were found to be statistically significant based on Kruskal Wallis Test ($P = 0.035$, $\chi2=8.63$, df = 3). **B. Overall Availability of each essential diagnostic test for TB.** In this figure, we present the overall availability of each test by combining data from all states and facility types, expressed as the percentage of facilities that offer the test. The results are shown as mean ± SD, and "N" represents the number of facilities surveyed. The differences in overall test availability were statistically significant according to the Kruskal-Wallis Test ($P= 0.029$, $\chi2=9.06$, df= 3). Availability of AFB smear test by microscopy and X-ray in PHCs **across states.** The figure shows the percentage availability of AFB and X-ray tests in PHCs across seven selected states. For states where more than one district was surveyed, district-level percentages were treated as replicates and presented as mean ± SD. Statistical analysis was not performed because in Himachal Pradesh (Sirmaur), Uttar Pradesh (Gautam Buddha Nagar), Rajasthan (Jaipur), and Haryana (Faridabad), only one district was surveyed in each state, so no replicates were available for comparison. **D. Availability of AFB smear test by microscopy, X-ray, and CBNAAT in CHCs across states and UT Delhi.** The figure shows the percentage availability of AFB, X-ray, and CBNAAT tests in CHCs across seven selected states and Delhi. For states or UT's where more than one district was surveyed, district-level percentages were treated as replicates and presented as mean ± SD. Statistical analysis was not performed because, in Himachal Pradesh (Sirmaur), Rajasthan (Jaipur), and Haryana (Faridabad), only one district was surveyed in each state; therefore, no replicates were available for comparison. **E. Availability of AFB smear test by microscopy, gram staining of pus cells by fluorescence microscope, X-ray, and CBNAAT in DH's across states and UT Delhi.** The figure shows the percentage availability of AFB, FM, X-ray, and CBNAAT tests in DHs across seven selected states and Delhi. State-wise values are shown as the percentage of DHs that have each test, and "N" represents the number of DH being surveyed. Statistical analysis was not performed because most districts have only one DH; therefore, no replicates were available, and only percentages could be reported.

4. Availability of CBC and CMP test panel across states and categories of healthcare facilities.

A. Availability of CBC and CMP test panel across state and UT Delhi: The figure shows the percentage availability of CBC and CMP test panels, as recommended in NEDL-2019 (Table 2), across all categories of healthcare facilities in the selected states and in the UT of Delhi. We calculated the

percentage of available tests out of the total recommended tests for CBC and CMP separately. Results are presented as mean ± SD, and "N" indicates the number of facilities surveyed. An independent sample t-test showed there is no statistical difference in the availability of CBC and CMP test panels across states (p-value =0.322, t = 1.026, df =14). **B. Availability of CBC and CMP test panel across categories of healthcare: The figure shows the percentage availability of CBC and CMP test panels, as recommended in NEDL-2019 (Table 2), across all categories of healthcare facilities by cumulating state-wise** values. We calculated the percentage of available tests relative to the total recommended tests for CBC and CMP, separately. Results are presented as mean ± SD, and "N" indicates the number of facilities surveyed. Mann-Whitney U test showed no statistically significant difference in the availability of CBC and CMP test panels across health care categories (p = 0.248, U = 4.0). **C. Availability of CBC and CMP test panel in SC's across three states:** The figure shows the percentage availability of CBC and CMP test panels recommended for SC's (Table 2), across Assam, Manipur, and Tripura. We calculated the percentage of available tests relative to the total recommended tests for CBC and CMP, separately. Results are presented as mean ± SD, and "N" indicates the number of facilities surveyed. Mann-Whitney U analysis showed there was no statistical difference in the availability of CBC and CMP test panels in SC's of these three states (p-value=0.121, Mann-Whitney U= 1.0). **D. Availability of CBC and CMP test panel in PHCs across seven states: The figure shows the percentage availability of CBC and CMP test panels recommended for PHCs (Table 2), across Assam, Manipur, Tripura, HP, UP, Rajasthan,** and Haryana. We calculated the percentage of available tests relative to the total recommended tests for CBC and CMP, separately. Results are presented as mean ± SD, and "N" indicates the number of facilities surveyed. From the independent sample t-test, we find that there is no statistical difference in the availability of CBC and CMP test panels in PHCs of select seven states (p-value=0.226, t = -1.28, df = 12). **E. Availability of CBC and CMP test panel in CHCs across seven states and UT of Delhi: The figure shows the percentage availability of CBC and CMP test panels recommended for CHCs (Table 2)** across Assam, Manipur, Tripura, HP, UP, Rajasthan, Haryana, and UT of Delhi. We calculated the percentage of available tests relative to the total recommended tests for CBC and CMP, separately. Results are presented as mean ± SD, and "N" indicates the number of facilities surveyed. Independent sample t-test showed there is no statistical difference in the availability of CBC and CMP test panels in CHCs of select regions (p-value = 0.149, t =1.526, df =14). **F. Availability of CBC and CMP test panel in DH's across six states and UT of Delhi:** The figure shows the percentage availability of CBC and CMP test panels recommended for DH's (Table 2) across Assam, Manipur, Tripura, HP, UP, Rajasthan, and UT of Delhi. We calculated the percentage of available tests relative to the total recommended tests for CBC and CMP, separately. Results are presented as mean ± SD, and "N" indicates the number of facilities surveyed. Mann-Whitney U analysis showed there is a statistical difference in the percentage availability of CBC and CMP test panels in DH's of select regions (p-value=0.004, Mann-Whitney U =7.0).

Reviewer #1 (Comments for the Author):

This study addresses an important and timely topic-TB diagnostic infrastructure gaps in India. The combination of national-level data extraction with field-level surveys provides valuable insights. However, several methodological, analytical, and presentation issues require attention.

1. The convenience sampling approach ("surveys were conducted only in locations where logistical support was available") introduces significant selection bias. The authors should explicitly acknowledge this as a limitation and discuss directionality of bias-facilities in accessible areas likely have better infrastructure than truly remote regions, suggesting the gaps reported may be underestimates.

Response: The selection of survey sites may appear to reflect logistical bias; however, it would not be accurate to classify these locations as non-remote or easily accessible simply because the survey was successfully conducted there. In the context of the North-East region of India, the selected sites are indeed remote and relatively difficult to access. The successful implementation of the survey in these areas was largely facilitated by local collaborators, who played a crucial role in securing the survey data. This demonstrates that, while such locations are challenging, they are not entirely inaccessible when appropriate local engagement is in place. Furthermore, the Foundation for Advancement of Essential Diagnostics (FAED), which is the primary contributor to this study, is based in North-East India. Several members of the study team, including those involved in manuscript preparation, are themselves from remote areas. This local presence and familiarity with the terrain and communities significantly contributed to the feasibility of conducting the survey in these settings.

While we agree with the reviewer that the site selection may reflect logistical bias, particularly given that our operational base is in these regions, our comprehensive nationwide TB Treatment Unit mapping and CDST Laboratory gap analysis still provide a larger national picture of TB preventative infrastructure in India. We also acknowledge that inclusion of all facilities across Assam, Tripura, and Manipur would have provided a more comprehensive and accurate estimation of diagnostic gaps in remote areas. But the present manuscript is intended to provide a first, preliminary snapshot of the diagnostic landscape in the region. In parallel, our survey is ongoing in other North-Eastern states and at the pan-India level; however, these efforts will require additional time, and the results are not yet ready to be included in this manuscript. We have now incorporated and highlighted this clarification in the Methods and Discussion sections of the revised manuscript (Refer to the Methods: Spectrum00703-26R1_RedlineTrackChange)

2. The highly uneven sampling across states (e.g., 159 facilities in Assam vs. 5 in Haryana; 5 districts in some states vs. 1 in others) limits meaningful inter-state comparisons. Consider removing state-level comparative statistics or reframing as exploratory/descriptive rather than comparative.

Response: We agree with the reviewer that this study reflects uneven sampling across districts and states, with relatively greater representation from Assam due to our operational presence in the region. This logistical and operational bias has now been explicitly acknowledged in the revised manuscript. We have also clarified that the primary purpose of this manuscript is to present a first, preliminary assessment of the availability of essential tuberculosis diagnostics, rather than to conduct state-wise statistical comparisons. The inclusion of state-wise data is intended solely to facilitate a clearer understanding of the preliminary status of diagnostic availability across different regions of India. We anticipate that our ongoing pan-India survey

will enable a more comprehensive and robust state-wise comparison in future publications. In line with the reviewer's suggestion, we have revised the manuscript text to avoid any unintended implication of formal state-wise comparisons.

3. Justification for state selection is absent. Why these seven states and Delhi? Do they represent geographic, demographic, or epidemiologic diversity? A rationale should be provided.

Response: The selection of study sites was guided by operational feasibility and existing field collaborations, as described in the Methods section of both the original and revised manuscripts (Refer to the Methods: Spectrum00703-26R1_RedlineTrackChange). However, in response to the reviewer's suggestion, we have further clarified the Discussion section (Refer to the Discussions: Spectrum00703-26R1_RedlineTrackChange) to indicate that the selected sites collectively represent a range of geographic regions and health system contexts. These include remote North-Eastern states, high-population northern states, and an urban metropolitan setting (Delhi), thereby capturing heterogeneity in healthcare infrastructure and access to diagnostic services.

4. The 18-month survey window (November 2023-April 2025) is problematic. Policy changes, equipment procurement, or infrastructure improvements during this period could affect comparability. Address whether any temporal adjustments were made.

Response:
We thank the reviewer for this important point. The data collection was conducted over about 18 months (November 2023 to April 2025), during which some changes in policies, equipment, or infrastructure may have occurred. As mentioned in the Methods section (Refer to the Methods: Spectrum00703-26R1_RedlineTrackChange), this was a cross-sectional survey based on facility visits, and no adjustments were made for time differences, as the aim was to provide a general snapshot of diagnostic availability rather than assess trends over time. We acknowledge that the longer survey period may affect comparability between sites. However, because changes in health systems usually occur gradually and at a much slower pace, we expect the overall findings to remain valid. We have now clarified this limitation of this study in the discussion section (Refer to the Discussions: Spectrum00703-26R1_RedlineTrackChange) of the revised manuscript. In particular, we would like to highlight to the reviewer that the survey is ongoing and that this limitation will be addressed more comprehensively in future publications.

5. The definition of "availability" needs clarification. Does this mean physical presence of equipment, functional equipment with reagents, or actual service provision? Prior literature demonstrates these differ substantially.

Response: In this study, "availability" was defined as the "actual provision of diagnostic services" at the facility at the time of assessment. This was determined through direct observation and verification with facility personnel using structured questionnaires. Availability was considered to include both the presence of functional diagnostic instruments and the necessary reagents at the facility. This definition has now been more explicitly described in the Methods section (Refer to the Methods: Spectrum00703-26R1_RedlineTrackChange) of the revised manuscript. In addition, detailed information about the questionnaire is provided in the supplementary materials.

6. **Private sector exclusion: Approximately 50% of Indian TB patients first seek care**

privately. The CDST gap calculations using only public facilities may significantly overestimate true gaps. Discuss this limitation explicitly.

Response: We thank the reviewer for this important observation. We agree that exclusion of private-sector diagnostic capacity would be an important limitation, particularly given that a substantial proportion of TB patients in India initially seek care in the private sector.

However, according to Suseela & Shannawaz (2023), there are about 2,000 private laboratories in India accredited by the National Accreditation Board for Testing and Calibration Laboratories (NABL), yet TB diagnostic services in the private sector remain limited. While there are 84 National Tuberculosis Elimination Program (NTEP)-certified Culture and Drug Susceptibility Testing Laboratories in the public sector, only 13 are in the private sector. These are part of our national CDST Laboratory Gap analysis, accounting for a total of 97 CDST Labs across India.

Additionally, while it could be true that about 50% of TB patients first seek care in the private sector, all drug susceptibility testing is still strictly maintained and monitored by the government/public healthcare under NTEP.

7. **Human resources: Equipment without trained personnel is non-functional. Staffing data (laboratory technicians, radiographers) should be collected or acknowledged as a limitation.**

Response: We thank the reviewer for this important observation. We agree that the availability of equipment alone does not ensure the delivery of functional services, as trained personnel are essential for effective laboratory operations. Data on staffing levels (e.g., laboratory technicians, radiographers) were initially part of the survey. However, during the survey, we observed that most facilities either had an in-house laboratory technician or shared one technician across two to three nearby facilities, depending on patient load. As a result, collecting detailed staffing information would have created a complex and cumbersome dataset, making it difficult to present clearly within the scope of this study.

Therefore, we focused on the key outcome of interest—the diagnostic services provided by each facility. Nonetheless, it was noted that the accuracy and reliability of test results were routinely monitored by the Medical Officer of the respective facility. Hence, it was acceptable to assume that if a facility provides a diagnostic service, the results generated are valid. This point has now been clearly explained in the Methods section (Refer to the Methods: Spectrum00703-26R1_RedlineTrackChange) of the revised manuscript.

8. Utilization data: Availability ≠ utilization. Were any data collected on actual test volumes or patient access?
Response:

We thank the reviewer for highlighting this important distinction. We agree that the presence of diagnostic services does not necessarily reflect their actual utilization by patients. However, the primary objective of this study was to assess the availability of essential diagnostic services at the facility level. Therefore, data collection was limited to documenting the presence of these services through field visits and structured questionnaires. We did not collect data on test volumes, frequency of use, patient footfall, or access barriers such as cost, referral delays, or patient pathways. As a result, our findings reflect infrastructural readiness rather than actual service utilization. It is also important to note that the government has defined standard population coverage for different levels of healthcare facilities (e.g., Sub-center: 3,000–5,000 population; PHC: 20,000–30,000; CHC: 80,000–120,000; District Hospital: ~2.5 million).

These norms provide a general approximation of expected service utilization at each level. Based on these standardized service coverage norms, we did not include real-time utilization data in this initial survey. However, we acknowledge that assessing actual utilization is important. This will be considered as part of a future, more detailed study. In the revised manuscript, we will include a statement on standard population coverage for each facility type (Refer Figure 1)

9.The abstract states "nationwide assessment of 327 healthcare facilities across seven states"-this is not truly "nationwide" given India has 28 states and 8 UTs. Revise language to "multi-state assessment" or similar.

Response:
We thank the reviewer for this observation. Accordingly, we have revised the abstract wording from “nationwide assessment” to “multi-state assessment” to more accurately reflect the study's coverage based on the facilities from which the primary survey data were generated. However, it should be noted that this study included a nationwide mapping of CDST laboratories and TB Units, which accurately reflects a nationwide assessment, as shown in Figure 2 and acknowledged by the reviewer as a nationwide data extraction study.

10. In methods, justification for choosing parametric vs. non-parametric tests is vague. Specify normality testing methods used.

Response: In this study, two normality tests are used: The Shapiro–Wilk Test and the Kolmogorov–Smirnov Test. In general, the null hypothesis states that the data are normally distributed. If the p-value is less than the chosen significance level (usually 0.05), it suggests that the data do not follow a normal distribution. These lines have been incorporated in the statistical analysis section of the method portion (Refer to the Methods: Spectrum00703-26R1_RedlineTrackChange).

11.The discussion is lengthy and somewhat repetitive of Results. Streamline to focus on interpretation, comparison with literature, and implications.

Response:
We thank the reviewer for this important comment and suggestion. We have significantly revised and rewritten the discussion to make it more interpretation-oriented rather than result-heavy.

12. Reference 2 is cited as "Global tuberculosis report 2025" but contains 2024 data-verify accuracy.

Response: We thank the reviewer for this. The reference has been corrected.

13. Several references are to government websites or portals without access dates or archived links

Response: We thank the reviewer for the comment. The suggested change has been incorporated in the revised manuscript.

Reviewer #2 (Comments for the Author):

Overall Assessment This manuscript addresses an important topic-the availability of TB diagnostic modalities across health care facilities in India. The findings have value, but the manuscript requires revision to improve clarity, accessibility, and alignment with the journal's audience.

Major Comments

1. Acronyms and region specific terminology The manuscript uses many acronyms and India specific terms (e.g., ICMR, PHC, CHC, lakhs, Nikshay) without defining them. For an international readership, all acronyms and local terminology should be introduced clearly at first use.

Response: We thank the reviewer for this important suggestion. We agree that clearly defining acronyms and region-specific terminology is essential to ensure accessibility for an international readership. In the revised manuscript, we have carefully reviewed the text and provided the full form of each abbreviation at its first occurrence.

2. Fit for the journal and special series While the work is not a microbiology study in the traditional sense, it does contribute to discussions on diagnostic access. Strengthening the link to the special series themes-point of care implementation, equitable access, and diagnostics for underserved populations-would help clarify its relevance.

Response: We thank the reviewer for this thoughtful and constructive suggestion. We agree that more explicitly aligning our study with the themes of the special series would strengthen its relevance to the journal's scope. In the revised manuscript, we have strengthened the framing in both the Introduction and Discussion sections to better highlight the study's contribution to the key theme of "laboratory equity". We have revised the manuscript to clearly position our work as a health systems and diagnostic access study, demonstrating how gaps in equitable implementation of essential diagnostics directly impact TB control efforts in underserved populations.

3. Missing conclusion. The manuscript does not include a conclusion section. A concise conclusion summarizing the key findings, their implications, and potential next steps would significantly strengthen the paper.

Response: We thank the reviewer for pointing this out. The conclusion has been included in the revised manuscript. Refer to the Conclusions: Spectrum00703-26R1_CleanVesion.

4. Systems level context A simple diagram outlining the tiers of the Indian health care system and the diagnostic services available at each level would greatly improve clarity for readers unfamiliar with the structure.

Response: We thank the reviewer for this great suggestion. In accordance with the reviewers' suggestion, we have included a diagram (Figure 1) that illustrates the different tiers of the Indian healthcare system and their corresponding standard population coverage. Details have been described in the figure legend to facilitate the reader's understanding.

5. Diagnostic capacity vs. equipment presence Reporting availability of equipment is helpful, but the manuscript should also address functionality, maintenance, and availability of trained personnel, as these factors determine true diagnostic capacity.

Response: We thank the reviewer for this important and insightful comment. We fully agree that the mere presence of diagnostic equipment does not equate to true diagnostic capacity, which depends on functionality, maintenance, and the availability of trained personnel. However, in the present study, our assessment focused on the final deliverable—namely, the provision of diagnostic tests—and was based on field surveys and structured questionnaires. As such, the survey reflects availability at a specific point in time and does not intend to overestimate service provision. That said, we acknowledge that facilities reporting availability may still face operational constraints that could limit or prevent the delivery of actual services. In response to the reviewer's suggestion, we have explicitly incorporated this clarification into the Methods section (Refer to the Methods: Spectrum00703-26R1_RedlineTrackChange) of the revised manuscript. We have also emphasized that our findings should be interpreted as representing infrastructural presence rather than fully functional diagnostic capacity. Furthermore, as this survey is ongoing, we plan to address this limitation in future analyses and publications.

6. Figures and supplementary materials Figures and supplementary items need clear labeling and referencing to guide the reader.

Response: We have made significant changes to the figure legends to ensure clearer, more concise descriptions.

7. Writing quality The manuscript would benefit from substantial editing for grammar, structure, and readability. The scientific insights are interesting-particularly regarding availability of AFB microscopy, X ray, CBNAAT, and FM-but the writing style and heavy use of unexplained acronyms obscure these strengths. A thorough language revision is recommended.

Response. We thank the reviewer for this positive comment and observation. We have made significant changes throughout the manuscript to improve on these points. A thorough language revision has been carried out as per the reviewers' suggestion. Refer to the Spectrum00703-26R1_RedlineTrackChange

Re: Spectrum00703-26R1 (Evaluating the nationwide availability of essential diagnostics for tuberculosis towards achieving a TB-free India)

Dear Dr. Mustafa A Barbhuiya:

Thank you for the privilege of reviewing your work. Below you will find my comments, instructions from the Spectrum editorial office, and the reviewer comments. In particular, please address the methodological queries from the reviewers with this next round of revisions.

Please return the manuscript within 60 days; if you cannot complete the modification within this time period, please contact me. If you do not wish to modify the manuscript and prefer to submit it to another journal, notify me immediately so that the manuscript may be formally withdrawn from consideration by Spectrum.

Revision Guidelines
To submit your modified manuscript, log into the submission site at https://spectrum.msubmit.net/cgi-bin/main.plex. Go to Author Tasks and click the appropriate manuscript title to begin. The information you entered when you first submitted the paper will be displayed; update this as necessary. Note the following requirements:

• Upload point-by-point responses to the issues raised by the reviewers in a file named "Response to Reviewers," NOT in your cover letter.
• Upload a compare copy of the manuscript (without figures) as a "Marked-Up Manuscript" file.
• Upload a clean .DOC/.DOCX version of the revised manuscript and remove the previous version.
• Each figure must be uploaded as a separate, editable, high-resolution file (TIFF or EPS preferred), and any multipanel figures must be assembled into one file.
• Any supplemental material intended for posting by ASM should be uploaded with their legends separate from the main manuscript. You can combine all supplemental material into one file (preferred) or split it into a maximum of 10 files with all associated legends included.

For complete guidelines on revision requirements, see our Submission and Review Process webpage. Submission of a paper that does not conform to guidelines may delay acceptance of your manuscript.

Data availability: ASM policy requires that data be available to the public upon online posting of the article, so please verify all links to sequence records, if present, and make sure that each number retrieves the full record of the data. If a new accession number is not linked or a link is broken, provide Spectrum production staff with the correct URL for the record. If the accession numbers for new data are not publicly accessible before the expected online posting of the article, publication may be delayed; please contact production staff (Spectrum@asmusa.org) immediately with the expected release date.

Publication Fees: For information on publication fees and which article types are subject to charges, visit our website. If your manuscript is accepted for publication and any fees apply, you will be contacted separately about payment during the production process; please follow the instructions in that e-mail. Arrangements for payment must be made before your article is published.

ASM Membership: Corresponding authors may join or renew ASM membership to obtain discounts on publication fees. Need to upgrade your membership level? Please contact Customer Service at Service@asmusa.org.

The ASM Journals program strives for constant improvement in our submission and publication process. Please tell us how we can improve your experience by taking this quick Author Survey.

Thank you for submitting your paper to Spectrum.

Sincerely,
Sophia Georghiou
Editor
Microbiology Spectrum

Reviewer #1 (Comments for the Author):

The manuscript is significantly improved and addresses majority of the concerns, except below two.

Justification for state selection: The Methods section should explicitly state the rationale for state selection, not just in the Discussion. While the Discussion (lines 447-452) notes that sites cover diverse regions, the Methods still mainly cites "operational feasibility and resource availability" (line 140), lacking a formal rationale. Strengthening justification in the Methods

rather than relying only on the Discussion.

18-month survey window: The Discussion does not clearly state that the 18-month survey window is a limitation, even though the authors said they would include this in their response letter. The Methods states the survey was "designed as a cross-sectional study at a single time point" (line 196), and the authors report no temporal adjustments. But the Discussion does not directly mention how the 18-month window could affect comparability. The cross-sectional design is noted, but the specific temporal limitation remains unaddressed. A sentence in the Discussion explicitly notes that the 18-month data collection window may introduce temporal heterogeneity, which should be added.

Reviewer #2 (Comments for the Author):

Minor Modifications Suggested

Reference to the "100-Day TB Elimination Mission"
The manuscript currently states: "The launch of the '100-Day TB Elimination Mission' by the Union Ministry of Health and Family Welfare on December 6, 2024."
Since the 100-day period has already elapsed, this phrasing should be updated to reflect its completion. I also encourage the authors to briefly comment on whether the mission achieved its stated objectives, and to clarify how the findings presented in this study relate to or inform the mission's outcomes.

Conclusion Requires Strengthening
The conclusion section would benefit from a clearer synthesis of the study's key findings and a more explicit articulation of their significance. Highlighting the main results and their implications for TB control, policy, or programmatic implementation will help reinforce the contribution of this work.

Reviewer #1 (Comments for the Author):

The manuscript is significantly improved and addresses majority of the concerns, except below two.

Comment 1: Justification for state selection: The Methods section should explicitly state the rationale for state selection, not just in the Discussion. While the Discussion (lines 447-452) notes that sites cover diverse regions, the Methods still mainly cites "operational feasibility and resource availability" (line 140), lacking a formal rationale. Strengthening justification in the Methods rather than relying only on the Discussion.

Response: We sincerely thank the reviewer for this insightful comment. Accordingly, we have revised the Methods section and added a detailed explanation regarding the rationale for state selection and the geographic diversity represented in the study (Lines 146–151 of the revised manuscript).

Comment 2: 18-month survey window: The Discussion does not clearly state that the 18-month survey window is a limitation, even though the authors said they would include this in their response letter. The Methods states the survey was "designed as a cross-sectional study at a single time point" (line 196), and the authors report no temporal adjustments. But the Discussion does not directly mention how the 18-month window could affect comparability. The cross-sectional design is noted, but the specific temporal limitation remains unaddressed. A sentence in the Discussion explicitly notes that the 18-month data collection window may introduce temporal heterogeneity, which should be added.

Response: We understand the reviewer's perspective on this observation. We respectfully disagree that the extended 18-month data collection period is a potential limitation affecting the comparability of findings across study sites. However, **to accommodate the limitation as understood in pure study design and statistical terms,** we have revised the Discussion section and explicitly acknowledged that, although each facility assessment was conducted as a cross-sectional evaluation at a single time point, the prolonged survey window may have introduced temporal heterogeneity due to changes in healthcare infrastructure, diagnostic capacity, resource availability, or policy implementation occurring during the study period. This objective limitation has now been incorporated in the revised manuscript (Lines 417–420).

Why would we disagree on the 18-month temporal variation study design limitation in this study?

The authors have been tracing and studying TB Diagnostics and TB Treatment infrastructure across India in the field for the past 6 years. The government's programmatic changes to upgrade infrastructure do not proceed quickly; the bureaucracy of policy change, approvals, and tenders plays an important role. One single CDST lab for TB Diagnostic setup, from approval to implementation and go-live for patient care in India under current Government regulations, will take up to 5 years. Note that TB Diagnostics and TB Treatment networks are fully regulated under the National TB Elimination Program, Government of India.

Reviewer #2 (Comments for the Author):

Minor Modifications Suggested

Comment 1: Reference to the "100-Day TB Elimination Mission"
The manuscript currently states: "The launch of the '100-Day TB Elimination Mission' by the Union Ministry of Health and Family Welfare on December 6, 2024."
Since the 100-day period has already elapsed, this phrasing should be updated to reflect its completion. I also encourage the authors to briefly comment on whether the mission achieved its stated objectives, and to clarify how the findings presented in this study relate to or inform the mission's outcomes.

Response: We sincerely thank the reviewer for this valuable suggestion. We agree that the original wording referred to the initiation of the 100 Day TB Elimination Mission and did not reflect its completion. To be fair and objective, based on our findings, it will not be possible to comment on 'pre' and 'post' 100-day TB Elimination Program impacts, as this study did not primarily ask that question. It is important to note that the 100 Day TB Elimination Program was primarily a TB Awareness program for the public, utilizing existing resources. The program did not focus on 'diagnostic infrastructure' and 'treatment infrastructure' improvement. The authors removed this mention, while follow-up future studies would help determine whether there was any tangible improvement in TB prevalence and drug resistance in India following the implementation of the 100 Day program.

Comment 2: Conclusion Requires Strengthening: The conclusion section would benefit from a clearer synthesis of the study's key findings and a more explicit articulation of their significance. Highlighting the main results and their implications for TB control, policy, or programmatic implementation will help reinforce the contribution of this work.

Response: Thank you again for helping the authors highlight the need for a more tailored conclusion. We have revised the conclusion section.

Re: Spectrum00703-26R2 (Evaluating the nationwide availability of essential diagnostics for tuberculosis towards achieving a TB-free India)

Dear Dr. Mustafa A Barbhuiya:

Your manuscript has been accepted, and I am forwarding it to the ASM production staff for publication. Your paper will first be checked to make sure all elements meet the technical requirements. ASM staff will contact you if anything needs to be revised before copyediting and production can begin. Otherwise, you will be notified when your proofs are ready to be viewed.

Data Availability: ASM policy requires that data be available to the public upon online posting of the article, so please verify all links to sequence records, if present, and make sure that each number retrieves the full record of the data. If a new accession number is not linked or a link is broken, provide production staff with the correct URL for the record. If the accession numbers for new data are not publicly accessible before the expected online posting of the article, publication may be delayed; please contact ASM production staff immediately with the expected release date.

Publication Fees: For information on publication fees and which article types have charges, please visit our website. We have partnered with Copyright Clearance Center (CCC) to collect author charges. If fees apply to your paper, you will receive a message from no-reply@copyright.com with further instructions. For questions related to paying charges through RightsLink, please contact CCC at ASM_Support@copyright.com or toll free at +1-877-622-5543. CCC makes every attempt to respond to all emails within 24 hours.

ASM Membership: Corresponding authors may join or renew ASM membership to obtain discounts on publication fees. Need to upgrade your membership level? Please contact Customer Service at Service@asmusa.org.

Embargo Policy: A press release may be issued as soon as the manuscript is posted on the Spectrum Latest Articles webpage. The corresponding author will receive an email with the subject line "ASM Journals Author Services Notification" when the article is available online.

The ASM Journals program strives for constant improvement in our submission and publication process. Please tell us how we can improve your experience by taking this quick Author Survey.

Thank you for submitting your paper to Spectrum.

Sincerely,
Sophia Georghiou
Editor
Microbiology Spectrum
