# Supplementary material for: Evaluating the nationwide availability of essential diagnostics for tuberculosis toward achieving a TB-free India
Source: Microbiol Spectr. 2026 Aug 11;14(9):e00703-26. doi: 10.1128/spectrum.00703-26 (PMC13532310; doi:10.1128/spectrum.00703-26)
Supplement: Supplementary file 2 [file spectrum.00703-26-s0005.docx]

**SUPPLEMENTARY FILE (S)**

**Supplementary Table 1.** Details of population, TB units, and CDST laboratories, including existing numbers, recommended requirements, ratios, and gaps. (A) Population as of 5 February 2025 (millions); (B) Population as of 19 April 2025 (lakhs); (C) TB unit requirement as per NTEP guidelines (1 per 200,000 population); (D) Recommended number of CDST laboratories; (E) Existing CDST laboratories; (F) Existing TB units; (G) Gap in TB units (recommended − existing); and (H) Gap in CDST laboratories (recommended − existing).

| **State & UT's** | **A.** | **B.** | **C.** | **D.** | **E.** | **F.** | **G** | **H.** |
| --- | --- | --- | --- | --- | --- | --- | --- | --- |
| A&N Islands | 0.4 | 4 | 2 | 0.08 | 0 | 9 | 0 | 0.08 |
| Andhra Pradesh | 53.58 | 535.8 | 268 | 10.72 | 4 | 363 | 0 | 6.72 |
| Arunachal Pradesh | 1.59 | 15.9 | 8 | 0.32 | 0 | 22 | 0 | 0.32 |
| Assam | 36.49 | 364.9 | 182 | 7.30 | 2 | 173 | 9 | 5.30 |
| Bihar | 131.04 | 1310.4 | 655 | 26.21 | 4 | 542 | 113 | 22.21 |
| Chandigarh | 1.26 | 12.6 | 6 | 0.25 | 1 | 5 | 1 | 0.00 |
| Chhattisgarh | 30.98 | 309.8 | 155 | 6.20 | 2 | 187 | 0 | 4.20 |
| DNHDD | 0.8 | 8 | 4 | 0.16 | 0 | 0 | 4 | 0.16 |
| Delhi | 22.28 | 222.8 | 111.4 | 4.456 | 4 | 46 | 65.4 | 0.456 |
| Goa | 1.59 | 15.9 | 7.95 | 0.318 | 1 | 19 | 0 | 0 |
| Gujarat | 73.51 | 735.1 | 367.55 | 14.702 | 3 | 329 | 38.55 | 11.702 |
| Haryana | 31.08 | 310.8 | 155.4 | 6.216 | 2 | 193 | 0 | 4.216 |
| Himachal Pradesh | 7.56 | 75.6 | 37.8 | 1.512 | 1 | 86 | 0 | 0.512 |
| Jammu & Kashmir | 13.83 | 138.3 | 69.15 | 2.766 | 1 | 81 | 0 | 1.766 |
| Jharkhand | 40.63 | 406.3 | 203.15 | 8.126 | 1 | 208 | 0 | 7.126 |
| Karnataka | 68.68 | 686.8 | 343.4 | 13.736 | 5 | 193 | 150.4 | 8.736 |
| Kerala | 36.11 | 361.1 | 180.55 | 7.222 | 2 | 56 | 124.55 | 5.222 |
| Ladakh | 0.3 | 3 | 1.5 | 0.06 | 0 | 5 | 0 | 0.06 |
| Lakshadweep | 0.07 | 0.7 | 0.35 | 0.014 | 0 | 1 | 0 | 0.014 |
| Madhya Pradesh | 88.99 | 889.9 | 444.95 | 17.798 | 6 | 347 | 97.95 | 11.798 |
| Maharashtra | 128.66 | 1286.6 | 643.3 | 25.732 | 15 | 476 | 167.3 | 10.732 |
| Manipur | 3.29 | 32.9 | 16.45 | 0.658 | 2 | 21 | 0 | 0 |
| Meghalaya | 3.43 | 34.3 | 17.15 | 0.686 | 1 | 22 | 0 | 0 |
| Mizoram | 1.26 | 12.6 | 6.3 | 0.252 | 0 | 13 | 0 | 0.252 |
| Nagaland | 2.28 | 22.8 | 11.4 | 0.456 | 0 | 22 | 0 | 0.456 |
| Odisha | 46.95 | 469.5 | 234.75 | 9.39 | 2 | 304 | 0 | 7.39 |
| Puducherry | 1.73 | 17.3 | 8.65 | 0.346 | 1 | 7 | 1.65 | 0 |
| Punjab | 31.19 | 311.9 | 155.95 | 6.238 | 2 | 164 | 0 | 4.238 |
| Rajasthan | 83.06 | 830.6 | 415.3 | 16.612 | 6 | 254 | 161.3 | 10.612 |
| Sikkim | 0.7 | 7 | 3.5 | 0.14 | 1 | 7 | 0 | 0 |
| Tamil Nadu | 77.39 | 773.9 | 386.95 | 15.478 | 7 | 413 | 0 | 8.478 |
| Telangana | 38.5 | 385 | 192.5 | 7.7 | 3 | 162 | 30.5 | 4.7 |
| Tripura | 4.2 | 42 | 21 | 0.84 | 1 | 21 | 0 | 0 |
| Uttar Pradesh | 241.27 | 2412.7 | 1206.35 | 48.254 | 11 | 1027 | 179.35 | 37.254 |
| Uttarakhand | 11.91 | 119.1 | 59.55 | 2.382 | 1 | 99 | 0 | 1.382 |
| West Bengal | 100.2 | 1002 | 501 | 20.04 | 5 | 431 | 70 | 15.04 |
| **SUM** | 1416.79 | 14167.9 | 7083.95 | 283.358 | 97 | 6308 | 1214.9 | 191.118 |

**Questionnaires for NEDL survey across different states in India**

In this survey, we assessed the availability of essential diagnostics for tuberculosis (TB), along with complete blood count (CBC) and comprehensive metabolic panel (CMP), across districts of Assam, Manipur, Tripura, Himachal Pradesh (HP), Uttar Pradesh (UP), Rajasthan, and the Union Territory of Delhi. The survey was conducted in accordance with the Indian Council of Medical Research (ICMR), Government of India recommendations under the National Essential Diagnostics List (NEDL), 2019. The complete list of recommended diagnostic tests for different categories of healthcare facilities is provided in Tables 2 (TB) and 3 (CBC & CMP). Data were collected through physical visits to randomly selected public healthcare facilities, including sub-centres (SCs), primary health centres (PHCs), community health centres (CHCs), and district hospitals (DHs), between November 2023 and April 2025. Facility staff were interviewed using structured questionnaires (Tables 2 and 3), and observations were documented. Availability of each diagnostic test was assessed using three criteria: (i) test reported to be conducted at the facility, (ii) availability of necessary equipment, and (iii) functional status of the equipment. A test was considered available only if all three criteria were met. Questions related to sample preparation and referral were not included for PHCs, CHCs, and DHs to avoid ambiguity, and only fully functional testing workflows—from sample processing to result generation—were considered, in line with NEDL recommendations. However, for SCs, questions on sputum collection and transport were included, as SCs are designated only for sample collection and referral for TB testing under NEDL guidelines. In total, 327 public healthcare facilities were surveyed: 159 in Assam, 19 in Manipur, 26 in Tripura, 57 in HP, 42 in UP, 14 in Rajasthan, 5 in Haryana and 5 in Delhi. The percentage availability of each diagnostic test was calculated as the proportion of facilities offering the test out of the total number of facilities of the same type surveyed.

**Supplementary Table 2. List of TB Essential Diagnostic Tests recommended for different Health Centres as per ICMR NEDL, 2019.**

| **Sl. No.** | **Category of Health Centres** | **Test Recommended** | **Specimen Type** | **Equipment Required** | **Presence/**  **Absence** |
| --- | --- | --- | --- | --- | --- |
| **1** | Village Level Health Centre | Sample collection for Acid- Fast Bacilli (AFB) Smear Microscopy Test | Sputum in disposable sterile containers | Sample to be sent to PHC for Microscopy |  |
| **2** | Sub-Centre  (SC) | Sample collection for AFB Smear Microscopy Test | Sputum in disposable sterile containers | Sample to be sent to PHC for Microscopy |  |
| **3** | Primary Health Centre (PHC) | AFB Smear Microscopy Test | Sputum in disposable sterile containers | Microscopy (ZN stain) |  |
|  |  | X-ray for TB Detection | Chest X-ray | Referral to nearest CHC |  |
|  |  | Mobile X-ray (We considered any form of X-ray facility) | | |  |
| **4** | Community Health Centre (CHC) | AFB Smear Microscopy Test | Sputum in disposable sterile containers | Microscopy (ZN stain) |  |
|  |  | Chip based Real time micro-PCR Test | Sputum in disposable sterile containers | Micro PCR |  |
|  |  | X-ray for TB Detection | Chest X-ray | X-ray Machine |  |
|  |  | Sample collection for Nucleic Acid  Amplification Test (NAAT) | Sputum in disposable sterile containers | Sample to be sent to DH for NAAT Test |  |
| **5** | Sub-District Hospital  (SDH) (Equivalent to CHC standard as per NEDL-2019 list) | AFB Smear Microscopy Test | Sputum, body fluids | Microscopy (ZN stain) |  |
|  |  | X-ray for TB Detection | Chest X-ray | X-ray Machine |  |
|  |  | Chip based Real time micro-PCR Test | Sputum in disposable sterile containers | Micro PCR |  |
|  |  | Sample collection for NAAT | Sputum in disposable sterile containers | Sample to be sent to DH for NAAT Test |  |
| **6** | District Hospital  (DH) | Sputum for AFB Microscopy Test  Pus for Gram Staining | Sputum, body fluids | Fluorescence Microscopy |  |
|  |  | Sputum Cytology | Sputum | Microscopy |  |
|  |  | X-ray for TB Detection | Chest X-ray | X-ray Machine |  |
|  |  | NAAT | Sputum | Truenat/Gene Xpert (PCR based) |  |
|  |  | Sample collection for TB culture (liquid) | Sputum, fluid etc. | Sample to be sent to nearest TB Lab/State TB Lab |  |

**Supplementary Table 3: List of CBC and CMP Essential Diagnostic Tests Recommended for Different Health Centres as per ICMR NEDL, 2019.**

| **Sl. No.** | **Category of Health Centres** | **Test Recommended** | **Presence/Absence** |
| --- | --- | --- | --- |
|  | SC | **CBC** | |
| 1 |  | Hemoglobin |  |
|  |  | **CMP** | |
| 1 |  | Blood sugar |  |
| 2 |  | Albumin |  |
|  | PHC | **CBC** | |
| 1 |  | Hemoglobin |  |
| 2 |  | Red blood cells count- |  |
| 3 |  | Total Leucocyte Count or WBC count |  |
| 4 |  | Platelets count |  |
|  |  | **CMP** | |
| 1 |  | Blood Sugar |  |
| 2 |  | Albumin |  |
|  | CHC | **CBC** | |
| 1 |  | Hemoglobin |  |
| 2 |  | Red Blood cells count |  |
| 3 |  | Total Leucocyte Count or WBC count |  |
| 4 |  | Platelets count |  |
| 5 |  | Hematocrit count |  |
| 6 |  | Mean Corpuscular Volume (MCV) |  |
|  |  | **CMP** | |
| 1 |  | Albumin |  |
| 2 |  | Blood sugar |  |
| 3 |  | Total bilirubin |  |
| 4 |  | Aspartate aminotransferase (AST) or SGOT |  |
| 5 |  | Alanine aminotransferase (ALT) or SGPT |  |
| 6 |  | Alkaline phosphatase |  |
| 7 |  | Creatinine |  |
| 8 |  | Blood urea |  |
| 9 |  | Sodium |  |
| 10 |  | Potassium |  |
| 11 |  | Calcium |  |
|  | DH | **CBC** | |
| 1 |  | Hemoglobin |  |
| 2 |  | Red Blood cells count |  |
| 3 |  | Total Leucocyte Count or WBC count |  |
| 4 |  | Platelets count |  |
| 5 |  | Hematocrit count |  |
| 6 |  | Mean Corpuscular Volume (MCV) |  |
|  |  | **CMP** | |
| 1 |  | Albumin |  |
| 2 |  | Blood sugar |  |
| 3 |  | Total bilirubin |  |
| 4 |  | Aspartate aminotransferase (AST) or SGOT |  |
| 5 |  | Alanine aminotransferase (ALT) or SGPT |  |
| 6 |  | Alkaline phosphatase |  |
| 7 |  | Creatinine |  |
| 8 |  | Blood urea |  |
| 9 |  | Sodium |  |
| 10 |  | Potassium |  |
| 11 |  | Calcium |  |
| 12 |  | Total protein |  |
| 13 |  | Blood Urea Nitrogen (BUN) |  |
| 14 |  | Chlorides |  |
| 15 |  | Blood Gas analysis or bi-carbonate |  |

**Supplementary Figures**

**Supplementary Figure 1.** Map showing survey locations in selected Indian states. The states—Assam, Tripura, Manipur, Himachal Pradesh (HP), Uttar Pradesh (UP), Rajasthan, Haryana, and Delhi—are highlighted in black on the inset map of India. Survey districts, marked in black on the respective state maps, include: Cachar, Karimganj (now Sribhumi), Hailakandi, Goalpara, and Kamrup Metropolitan (Assam); Imphal East and Imphal West (Manipur); North Tripura, Unakoti, South Tripura, and West Tripura (Tripura); Sirmaur (HP); Aligarh, Budaun, Kasganj, and Gautam Buddh Nagar (GBN) (UP); and Jaipur (Rajasthan). The Delhi National Capital Region (Delhi-NCR) was also included.

**Supplementary Figure 2.** District-wise availability of essential diagnostics (ED) for TB diagnosis. (A) Percentage availability of facilities with complete ED for TB diagnosis across districts. Data are expressed as mean ± SD; N indicates the total number of healthcare facilities surveyed in each district across all facility types. No statistical analysis was performed due to unequal distribution of facility types across districts, as only a single category of healthcare facility was surveyed in some districts due to resource constraints. (B) District-wise availability of ED in sub-centres (SCs), expressed as percentage; N represents the total number of SCs surveyed per district. (C) District-wise availability of ED in primary health centres (PHCs), expressed as percentage; N represents the total number of PHCs surveyed per district. (D) District-wise availability of ED in community health centres (CHCs), expressed as percentage; N represents the total number of CHCs surveyed per district. No statistical analysis was performed for panels B–D due to limited sample sizes.

**Supplementary Figure 3.** District-wise availability of individual essential diagnostic (ED) tests for TB diagnosis. (A) Percentage availability of individual ED tests at primary health centres (PHCs) across districts. Data are expressed as percentages; N indicates the total number of PHCs surveyed in each district. A Mann–Whitney U test showed a significant difference in the availability of AFB smear microscopy and X-ray across districts (p = 0.004; U = 20.5). (B) Percentage availability of ED tests at community health centres (CHCs) across districts. Data are expressed as percentages; N represents the total number of CHCs surveyed per district. A Kruskal–Wallis test showed a significant difference in the availability of AFB smear microscopy, X-ray, and CBNAAT across districts (p = 0.001; χ² = 14.198; df = 2).

**Supplementary Figure 4.** District-wise availability of CBC and CMP test panels. (A) Percentage availability of CBC and CMP panels in sub-centres (SCs) across districts. Data are presented as mean ± SD; N indicates the number of SCs surveyed per district. No significant difference was observed among districts (Mann–Whitney U test; p = 0.071, U = 4). (B) Percentage availability of CBC and CMP panels in primary health centres (PHCs) across districts. Data are presented as mean ± SD; N represents the number of PHCs surveyed per district. A significant difference was observed among districts (Mann–Whitney U test; p = 0.010, U = 43). (C) Percentage availability of CBC and CMP panels in community health centres (CHCs) across districts. Data are presented as mean ± SD; N indicates the number of CHCs surveyed per district. A significant difference was observed among districts (Mann–Whitney U test; p = 0.010, U = 95).
